# Serum fatty acid chain length associates with prevalent symptomatic end-stage osteoarthritis, independent of BMI

**DOI:** 10.1038/s41598-020-71811-3

**Published:** 2020-09-22

**Authors:** J. M. T. A. Meessen, F. Saberi-Hosnijeh, N. Bomer, W. den Hollander, J. G. van der Bom, J. A. van Hilten, W. E. van Spil, C. So-Osman, A. G. Uitterlinden, M. Kloppenburg, R. G. H. H. Nelissen, C. M. van Duijn, P. E. Slagboom, J. B. J. van Meurs, I. Meulenbelt

**Affiliations:** 1 Department of Orthopaedics, Leiden University Medical Center, Leiden, The Netherlands; 2 Molecular Epidemiology, Department of Biomedical Data Sciences, Leiden University Medical Center, Postzone S-05-P, Einthovenweg 20, 2333 ZC Leiden, The Netherlands; 3 Department of Internal Medicine, Erasmus Medical Center, Rotterdam, The Netherlands; 4 Department of Immunology, Erasmus Medical Center, Rotterdam, The Netherlands; 5 Unit of Transfusion Medicine, Sanquin Blood Bank, Leiden, The Netherlands; 6 Department of Rheumatology and Clinical Immunology, University Medical Center Utrecht, Utrecht, The Netherlands; 7 Department of Rheumatology, Leiden University Medical Center, Leiden, The Netherlands; 8 BBMRI Metabolomics Consortium the Netherlands, Leiden, The Netherlands; 9 Department of Epidemiology, Erasmus Medical Center, Rotterdam, The Netherlands

**Keywords:** Metabolomics, Biomarkers, Rheumatology

## Abstract

Higher body mass index (BMI) is associated with osteoarthritis (OA) in both weight-bearing and non-weight-bearing joints, suggesting a link between OA and poor metabolic health beyond mechanical loading. This risk may be influenced by systemic factors accompanying BMI. Fluctuations in concentrations of metabolites may mark or even contribute to development of OA. This study explores the association of metabolites with radiographic knee/hip OA prevalence and progression. A ^1^H-NMR-metabolomics assay was performed on plasma samples of 1564 cases for prevalent OA and 2,125 controls collected from the Rotterdam Study, CHECK, GARP/NORREF and LUMC-arthroplasty cohorts. OA prevalence and 5 to 10 year progression was assessed by means of Kellgren-Lawrence (KL) score and the OARSI-atlas. End-stage knee/hip OA (TJA) was defined as indication for arthroplasty surgery. Controls did not have OA at baseline or follow-up. Principal component analysis of 227 metabolites demonstrated 23 factors, of which 19 remained interpretable after quality-control. Associations of factor scores with OA definitions were investigated with logistic regression. Fatty acids chain length (FALen), which was included in two factors which associated with TJA, was individually associated with both overall OA as well as TJA. Increased Fatty Acid chain Length is associated with OA.

## Introduction

Osteoarthritis (OA) is very prevalent, age-related, progressive degenerative disease of articular joints and one of the leading causes of disability and pain worldwide^1^. Due to ageing, increased longevity, and increasing obesity of the population, the OA incidence is expected to rise even further^2–4^. Epidemiological studies have shown that unfavourable metabolic parameters, such as high body mass index (BMI), waist-hip ratio and proportion of fat mass associated with OA onset and its severity^5–11^. Conversely, weight loss has been shown to significantly relief pain while increasing physical function of people with knee OA^12–15^. Notably, these associations have been found for both the weight-bearing joint of the knee and non-weight bearing joints of the hand, suggesting a connection between OA and obesity beyond increased loading^16–18^. In line with this, OA has been shown to associate with classical markers of poor metabolic health such as increased circulating levels of low density lipoprotein (LDL) and cholesterol^19,20^. However, this connection remains controversial for the hip joint^21^.

Recently, studies investigated metabolic profiles to identify biomarkers of poor metabolic health in association with OA and its progression. Few small studies were performed into circulating serum metabolomics biomarkers, identifying that ratios of amino acids such as valine and xleucine to histidine and alanine, arginine, aspartate, glutamate and proline are associated with OA^22–25^. More studies have been performed using synovial fluid which have confirmed the some of the aforementioned amino acids, as well as identifying altered energy-, collagen-, fatty acid-, glycerolipid-, oxidative stress and nitric oxide-metabolisms^26–28^ suggesting an altered metabolic state of the synovial fluid in OA. However, these hallmarks of the metabolic syndrome (MS) should also be present in serum as MS affects not only the synovium but also the whole body. In addition, consensus on biomarkers is lacking and the sample size of these studies was generally small. Warranting the need to perform large-scale studies to elucidate the link between metabolites and OA.

Therefore, in the current study, we set out to conduct the first large scale metabolomics assessment including metabolites ranging from amino acids to lipids to fatty acids in over four thousand cases and controls. We aim to identify metabolic signatures of serum by including 2,125 controls and 2,372 OA cases among Dutch cohorts participating in the Biobanking and BioMolecular resources Research Infrastructure consortium (BBMRI metabolomics consortium)^29^.

## Results

Levels of 231 serum metabolic traits (Supplementary Table 2) were obtained on the NMR (Nightingale) platform. Of the 231 metabolites, 4 [conjugated linoleic acid (CLA), the ratio of CLA to total fatty acids (CLAFA), diglycerids (DAG) and ratio DAG to triglycerids (DAG/TG)] had more than 5% of the measurements below the detection limit and were excluded from analysis. Analyses of the 227 remaining metabolites was performed in 2,125 controls and 1,556 OA cases from 4 independent studies participating in the Biobanking and BioMolecular resources Research Infrastructure consortium (BBMRI metabolomics consortium, Supplementary Table 1). Baseline characteristics of cases and controls are shown in Table 1.

**Table 1:** Baseline characteristics of subjects in the cross-sectional analyses.

	Controls	Overall OA^A^			TJA		
	N = 2,125	All N = 1556	Hip N = 736	Knee N = 990	All N = 640	Hip N = 447	Knee N = 199
Female (%)	1,301 (61%)	1,554 (68%)	482 (66%)	700 (71%)	638 (70%)	313 (70%)	142 (72%)
Age (SD)	66.1 (10.1)	70.9 (10.3)	69.6 (11.9)	72.5 (8.6)	68.9 (12.2)	68.0 (13.1)	71.0 (9.3)
BMI (SD)	26.5 (3.9)	28.1 (4.5)	27.5 (4.4)	28.7 (4.5)	28.2 (4.6)	27.6 (4.6)	29.8 (4.1)

*OA* osteoarthritis, *TJA* Total joint arthroplasty.

^A^Overall Radiographic OA as defined by KL-score ≥ 2 and/or total joint arthroplasty.

### Principal component analysis (PCA)

To reduce data dimensions of the correlated metabolites, PCA analyses was performed. In a PCA analysis the underlying the structure of correlation between individual metabolites is taken into account and merged into independent factors. As shown in Supplementary Table 3, 23 factors with an eigenvalue of ≥ 1, accounting for 91,4% of the total variance were identified. Indicating that in the 227 assessed metabolites, 23 independent correlation structures are present. Prior to the analyses for OA status, possible confounding effects of cohorts were investigated by analysing the principal components as function of controls of each cohort while adjusting for age, sex, BMI and fasting status. Hereto four factors (factor-2, factor-10, factor-13 and factor-16), accounting for 28.06% of the variance were removed from the analyses.

### Cross-sectional analyses of prevalent overall OA

After adjusting for age, gender BMI and multiple testing, we identified five factors (factor-4, factor-11, factor-15 factor-19, and factor-20) that were significantly associated to overall OA as defined by a KL score of ≥ 2 or having a total joint arthroplasty in hip and/or knee joints (Table 2).

**Table 2:** Cross-sectional analyses of factors with prevalent overall osteoarthritis and total joint arthroplasty.

Factor	Controls	Overall OA^A^ (N = 1556)			TJA (N = 640)		
		OR	95%CI	*P*_adjusted_^B^	OR	95%CI	*P*_adjusted_^B^
1	2,125	0.96	0.88–1.04	NS	0.79	0.70–0.89	2.6 × 10^–3^
3	2,125	0.98	0.90–1.07	NS	1.10	0.96–1.25	NS
4	2,125	1.25	1.15–1.36	3.0 × 10^–6^	1.44	1.26–1.64	1.2 × 10^–6^
5	2,125	1.01	0.93–1.09	NS	0.99	0.86–1.13	NS
6	2,125	1.04	0.96–1.13	NS	1.20	1.07–1.34	4.5 × 10^–2^
7	2,125	0.91	0.85–0.99	NS	0.84	0.74–0.95	NS
8	2,125	1.03	0.94–1.12	NS	1.12	0.96–1.30	NS
9	2,125	1.03	0.94–1.11	NS	0.93	0.82–1.06	NS
11	2,125	0.87	0.80–0.93	4.3 × 10^–3^	0.78	0.69–0.88	1.7 × 10^–3^
12	2,125	1.00	0.92–1.08	NS	1.05	0.93–1.19	NS
14	2,125	1.02	0.94–1.10	NS	1.04	0.92–1.18	NS
15	2,125	0.84	0.77–0.91	3.4 × 10^–4^	0.86	0.75–0.97	NS
17	2,125	1.02	0.95–1.11	NS	1.36	1.21–1.54	1.2 × 10^–5^
18	2,125	0.89	0.82–0.96	NS	0.90	0.79–1.04	NS
19	2,125	0.75	0.69–0.82	7.6 × 10^–9^	0.64	0.56–0.74	9.6 × 10^–9^
20	2,125	0.85	0.78–0.93	5.0 × 10^–3^	0.91	0.79–1.04	NS
21	2,125	0.93	0.86–1.01	NS	0.99	0.87–1.13	NS
22	2,125	1.12	1.04–1.21	NS	1.50	1.33–1.7	2.2 × 10^–9^
23	2,125	1.03	0.95–1.11	NS	1.51	1.33–1.72	9.0 × 10^–9^

*OA* osteoarthritis, *TJA* Total joint arthroplasty, *OR* Odds Ratio, *95%CI* 95% Confidence interval.﻿

^A^Overall Radiographic OA as defined by KL-score ≥ 2 and/or total joint arthroplasty. ^B^Adjusted for age, gender, BMI and multiple testing.

Of these, most significantly associated to overall OA were factor-4 with OR 1.25 95% CI 1.15–1.36, *P* < 0.001 and factor 19 OR 0.75, 95% CI 0.69–0.82, *P* < 0.001. In order to assess the type of biology that each factor represents, we looked at the individual correlated metabolites for each factor. Based on the metabolites that, among others, load on these factors (Supplementary Table 2) we conclude that factor-4 represents mainly the medium and large triglycerides to total lipids ratio and factor 19 particularly the amino acids glutamine and histidine). The metabolites that represent factor-11 and factor-20 are different types of fatty acid ratios and 3-hydroxybutyrate and citrate, respectively. Different cholesterols represent factor-15. Associations separate for hip and knee OA were comparable (Supplementary Table 4).

### Cross-sectional analyses total joint arthroplasty

To investigate associations among the part of the cases representing patients with a total joint arthroplasty surgery, and therefore symptomatic end-stage disease, we performed similar cross-sectional analyses with total joint arthroplasty. Even though these analyses had less power than the cross-sectional analyses for overall OA due to a smaller sample size, the effect sizes were more pronounced and the levels of significance were comparable to the overall OA analyses. After adjusting for age, gender, BMI and multiple testing, we identified eight factors (factor-1, factor-4, factor-6, factor-11, factor-17, factor-19, factor-22, and factor-23) that were significantly associated to patients with joint arthroplasty of knee and/or hip (Table 2).

Notably, factor-1, factor-6, factor-17, factor-22, and factor-23 did not show association to overall OA and may thus be specifically associated with arthroplasties. These associations were slightly more prominent for arthroplasty in the knee as compared to hip arthroplasty (Supplementary Table 5). Based upon the metabolites loading on these factors we conclude that factor-1 is mainly representative of the LDL/VLDL metabolism, factor -6 represents cholesterol(esters), factor-17 and factor-23 represent different aspects of the energy cycle and factor-22 represents fatty acid make-up.

To further assess the confounding effect of fasting-status, we performed additional analyses in- and excluding adjustment for fasting. As shown in Supplementary Table 6, the association of the factors with OA remained consistent with and without an adjustment for fasting. Also, upon splitting the cohort in those with BMI < 30 and BMI ≥ 30, most factors showed similar odds ratio’s between the two groups. Notably, the effect of factor 4 and 17 was slightly larger in the more obese group while factor 19 was stronger in those with BMI < 30. Factor 22 was the most robust with comparable Odds Ratio’s between the two groups (Supplementary Table 7).

### Cross-sectional analyses of individual metabolites

Factors 1, 4, 6, 11, 17, 19, 22 and 23, which were associated with arthroplasty, were selected for further in-depth analysis. To investigate whether any of the individual metabolites within the identified factors drive the associations, we assessed the association with the individual metabolites significantly represented in these factors (loading on the factor > 0.4 or < − 0.4). As shown in Supplementary Table 8, the majority of the individual metabolites in the factors were associated to a similar extent (OR and P-value) to arthroplasty and as such reflect high correlation between the individual metabolite features within a factor. A notable exception, however, is FALen (Fatty Acid chain Length) represented in factor-1 and factor-22. The association with the individual FALen metabolite stands out as it appears to give the strongest and most specific association with TJA (OR 1.83, 95% CI 1.64–2.05, P < 0.001, see also Table 3. In order to assess any differences in the association of FALen and joint site, we have assessed the association of FALen with different joint sites and phenotypes. As shown in Table 3, FALen was significantly associated with all included phenotypes, with the strongest association found for knee arthroplasty. Additionally, as shown in Supplementary Table 7, factor 22 driven by FALen is stable across the different BMI strata. As statins might affect the concentrations of metabolites, we performed sensitivity analyses to assess their influence on our outcomes. The use of statins was known for all included studies except CHECK. Sensitivity analysis revealed only minor changes in the effect sizes. Similarly, upon in- and excluding an adjustment for statins in the analyses, the Odds Ratio’s changed marginally.

**Table 3:** Cross sectional analyses of metabolite FALen representing fatty acid chain length with different OA types.

Phenotype	N_controls_	N_cases_	FALen		
			OR	95% CIL	*P*_adjusted_^A^
All OA	2,125	1556	1.26	1.16–1.36	3.6 × 10^–8^
Hip OA	2,125	736	1.39	1.26–1.54	8.2 × 10^–10^
Knee OA	2,125	990	1.15	1.05–1.25	2.1 × 10^–2^
Arthroplasty	2,125	640	1.83	1.64–2.05	1.0 × 10^–320^
THA	2,125	447	1.73	1.52–1.98	3.2 × 10^–15^
TKA	2,125	199	2.10	1.75–2.51	5.6 × 10^–15^

*FALen* fatty acid chain length, *THA* total hip arthroplasty, *TKA* total knee arthroplasty.

^A^Adjusted for age, gender, BMI and multiple testing.

### Progression of OA

As FALen showed strong associations with TJA as well as with overall OA (OR 1.26, 95% CI 1.16–1.36, P < 0.001), we next explored its association with the progression of OA. Progression data was available for N = 2009 (N = 721 non-progressors and N = 305 progressors in the Rotterdam Study and N = 522 non-progressors and N = 130 progressors in CHECK) (Materials and Methods). As shown in (Supplementary Fig. 1) and despite the high and consistent cross sectional association the individual metabolite FALen was not associated to OA progression.

## Discussion

Serum metabolomic assays of hip and knee OA cases and controls were assessed by means of the Nightingale ^1^H-NMR platform, resulting in 227 different metabolite measurements. PCA-analysis identified 23 factors explaining 71.9% of variance, of which 19 were eligible for further regression analyses. Eight factors (factor-1, factor-4, factor-6, factor-11, factor-17, factor-19, factor-22 and factor-23) were associated with total joint arthroplasty independent of age, sex and BMI. Assessing the association of individual metabolites of these factors with TJA revealed a highly significant association between Fatty Acid Chain Length (FALen) and Any OA or TJA (P < 0.001). Upon splitting the phenotypes by joint site (hip or knee) consistent, significant positive association was found for FALen and all four additional phenotypes. As such, this study places a composite score of fatty acid length at the heart of the link of osteoarthritis and metabolites. This study showed that FALen has a strong association with end stage OA as represented by overall radiographic OA (OR 1.26) but particularly to joint arthroplasty (OR 1.83).

Recently, the comprehensive review by Harasymowicz et al*.* described the many ways how fatty acids influence not only the cartilage, but also bone and synovial fluids in the joint^30^. Specifically, they describe that longer chain saturated fatty acids have a negative impact on the homeostasis of cartilage and chondrocyte metabolism. In addition, long-chain SFA inhibits osteoclast-genesis, whereas shorter-chain SFA enhance osteoclast-genesis^30,31^. Another recent study has shown that longer-chain dietary fatty acids in rats induce both metabolic syndrome and changes in the knee joint resembling OA^32^, all confirming our finding that longer chains of the fatty acid are associated with end stage, symptomatic OA. Fatty acids are known to play a role in a broad range of cardiovascular diseases as well as to the immune system, which might hint that there is a more generic pathway underlying the association of OA and fatty acid make up^33,34^. This finding was supported by the study of Senol et al*.* in OA patients which demonstrated that there are differences in the metabolic pathways of fatty acid biosynthesis pathways between OA patients and controls^35^. Given these studies, we advocate that the length of the Fatty Acid Chain is underlying the risk of OA susceptibility by directly affecting chondrocyte energy metabolism. Indeed, pathways which are activated during high concentrations of fatty acid in blood such as mTOR and AMPK are found to be associated with knee OA in mouse. Leading to increased apoptosis in mouse knee and aging of the murine cartilage^36^. Reduced AMPK is associated with a loss of mitochondrial function, which in turn leads to ageing of the cartilage^37^. Again, this indicates a more generic pathway underlying the association of fatty acids with OA^38^.

We did not demonstrate any association of FALen with OA progression. This may be explained by the fact that OA progression was defined by KL-scores alone, as such differ as the symptomatic end stage OA of patients undergoing arthroplasty. For that matter, in Supplementary Fig. 1 it is suggested that the effect for FALen and OA-progression is larger in the CHECK cohort as compared to that in the Rotterdam study as inclusion criteria of CHECK patients were based on symptoms and in the Rotterdam Study based on radiographic OA alone.

FALen was present in both Factor-1 and Factor-22, the first explaining a large part of the variance in data (31.25%), whereas factor-22 explains 0.49%. The fact that FALen is present in both and that both are significantly associated with TJA, shows that it is worthwhile to explore also small factors. Notably, two other metabolites that were represented in factor-22, hence showing an underlying correlation structure to FALen, were Degree of Unsaturation (UnsatDeg) and ratio of saturated fatty acids (SFA FA). Nonetheless, the absence of association of these metabolites to TJA (Supplementary Table 8) confirms that the found association of Factor-22 to TJA (OR 1.50, 1.33–1.70) is almost totally attributional to fatty acid chain length (FALen; OR 1.83, 1.64–2.05) and not to fatty acid saturation (UnsatDeg OR 1.12, 95% CI 0.99–1.28 and SFA FA OR 0.95 95% CI 0.84–1.07).

The fact that FALen is also present in Factor-1, alongside many other metabolites in the LDL/VLDL range, shows that besides its own specific interaction with OA, FALen plays a role through lipid-transportation, as is reflected by Factor-1^30^. This double presence shows the highly correlated interplay of metabolites and reflects the fact that the length of a fatty acid chain length alters the characteristics of the fatty acid, influencing the metabolism. Although studies have shown that the link between OA and obesity is more often present in knee OA, we found in this study that FALen is associated to both knee and hip OA, with the strongest association found for knee replacement surgery. This may indicate a more general link between OA and fatty acid length, beyond obesity and knee OA which is also supported by the stable associations of factor 22 (driven by FALen) across the BMI strata (Supplementary Table 7).

In this study, we choose to differentiate between patients who underwent total hip or knee arthroplasty from patients with radiographic signs of OA. This because arthroplasty-patients are essentially in a different (end)stage of the disease, and an additional incentive to undergo joint replacement surgery has taken place. In contrast, patients with radiological OA represent a range of patients with symptoms who may not (yet) be severe enough for an indication for arthroplasty. The fact that we observed stronger associations with arthroplasty patients, in spite of lower power, supports the view that deviation of metabolic profiles gradually accompanies aspects of the OA process towards a joint replacement surgery. However, disentangling the relationship between FALen, OA symptoms, and joint replacement requires additional in depth investigation preferably in studies in which symptoms, radiographic OA and the incentive towards a joint replacement surgery is accurately documented.

A strength of our study was the relatively large sample size as compared to reported studies. The combination of different studies to reach more power also meant that we incorporated some studies with only cases or controls, raising the chance of cohort effects. However, we did assess the presence of cohort effects within the control group, where no differences should be present between the cohorts as all samples are OA-free. Only factors free of cohort effects were included in our analyses. We adjusted our analyses for baseline-BMI; however, we cannot exclude bias in our findings due to the effect of weight loss or gain during follow-up. Our study included both fasted and nonfasted samples while metabolites may be sensitive to fasting status, therefore we assessed the influence of including a correction or fasting status to assess dietary influences. Despite the fact that food intake influences metabolite patterns the association with OA remained untouched^39^. We demonstrated only marginal changes, i.e. effect sizes were very similar.

The current paper is to our knowledge the first large scale hypothesis free approach in search for metabolites that associate to OA in a cross-sectional design. Future research should particularly focus on replication of the found results and, if this succeeds, further elucidate the mechanisms behind the association of fatty acid chain length and OA.

In conclusion, the current study identified a number of metabolic factors associated with OA, independent of BMI. This indicates that there is an altered metabolic state in patients with OA as compared to controls without OA. This is another token that OA should be seen as a component of poor metabolic health and that recording systemic factors of health status in older people may represent an informative marker of OA risk.

## Methods

### Study populations

Metabolic signatures were assessed in N = 2,125 controls and N = 1,556 cases from 4 Dutch cohorts being Cohort Hip & Cohort Knee (CHECK, N = 864), Genetics, Arthrosis and Progression (GARP-study, N = 216) with its NORmal REFerence Controls (NORREF, N = 34), Leiden University Medical Centre (LUMC) Arthroplasty studies (LUMC-cohorts N = 455) and the Rotterdam study (N = 2,112) that participated in the Biobanking and BioMolecular resources Research Infrastructure consortium (BBMRI metabolomics consortium)^29^. Distribution of cases and controls across these studies is depicted in Supplementary Table 1. Informed consent was obtained from all included participants. The experiment protocol was followed according to the Declaration of Helsinki and good clinical practice. In addition, this study was assessed by the steering committees of each included study and the Medical Ethics Committees of all participating centres approved the study [Albert Schweitzer Hospital (Dordrecht), Atrium Medisch Centrum (Heerlen), Canisius-Wilhelmina Ziekenhuis (Nijmegen), Erasmus Medical Center (Rotterdam), Groene Hart Hospital (Gouda), Isala Klinieken (Zwolle), Kennemer Gasthuis (Haarlem), Leyenburg ziekenhuis (The Hague) Leids Universitair Medisch Centrum (Leiden), Maastricht University Medical Center (Maastricht), Martini Ziekenhuis (Groningen), Medisch Centrum Alkmaar (Alkmaar), Medisch Centrum Haaglanden (The Hague), Medisch Spectrum Twente (Enschede), Reade (Amsterdam), Reinier de Graaf Groep (Delft), Rijnland ziekenhuis (Leiderdorp), Slotervaart Hospital (Amsterdam), St. Antonius ziekenhuis (Nieuwegein), St. Joseph Ziekenhuis (Veldhoven), St. Maarten's Gasthuis (Venlo) Tweesteden Ziekenhuis (Tilburg), Universitair Medisch Centrum Utrecht (Utrecht), Ziekenhuis Rijnstate (Arnhem)].

*CHECK* is a prospective, 10-year follow-up, observational cohort study of 1,002 people aged between 45 and 65 years at the time of inclusion, with pain in their knee(s) and/or hip(s), who had never or not longer than 6 months ago visited a general physician for these complaints^40^. Blood samples were taken non-fasted. Hip and knee radiographs were scored pairwise according to the Kellgren & Lawrence (KL) scoring system^41^. When scored pairwise, these people did not have obvious radiographic knee or hip OA at baseline (i.e. KL = 0 or 1)*.* As such, these persons were considered controls for the cross-sectional analyses on OA prevalence at baseline. For the current study we included 864 persons from the CHECK Cohort.

*GARP/NORREF study:* The GARP cohort (N = 216) consists of patients with advanced radiographic OA at two or more joint sites of hand, spine, knee or hip. Matched to the GARP study, a normal reference control group (NORREF) was collected using the same protocol and included in this study as controls (N = 34)^42–44^. In the current study baseline non-fasting plasma samples were included.

*LUMC cohorts:* The LUMC arthroplasty studies (N = 455) consist of participants of the “Research Arthritis and Articular Cartilage” (RAAK), “Transfusion induced complications = Transfusion associated complications?” (Tactics) (NTR309) and “Transfusie op Maat” (TOMaat) (NTR303) studies^45,46^. These cross-sectional studies included OA patients who received THA or TKA. Blood samples were collected during surgery while patients were fasted.

*Rotterdam Study:* The Rotterdam Study (RS) is a large prospective population-based cohort study of men and women aged 55 years and older in the municipality of Rotterdam, the Netherlands. The study design and rationale are described elsewhere in detail^47^. In summary, the objective of the study is to investigate determinants, incidence and progression of chronic disabling diseases in the elderly. Baseline measurements were obtained through a home interview and visits to the research centre for physical examinations and imaging and laboratory assessments, blood samples were taken while patient was fasted. The present study includes 2,112 baseline participants from RS-I (Ergo 4). The study included both individuals with and without OA with a mean follow-up time of 6.5 year. Blood was sampled nonfasted. The study included both individuals with and without OA at baseline with mean follow-up time of 6.5 years.

### Definitions of OA

Prevalent radiographic OA was (N = 1556) defined as either having a KL score of ≥ 2 in hip and/or knee at baseline or having THA or TKA for primary OA^41^. THA/TKA patients (N = 640) were also assessed separately. Controls (N = 2,125) had no radiographic hip and knee OA (KL < 2) and were selected from the Rotterdam Study, CHECK and NORREF as described above. Controls were selected from CHECK and the Rotterdam Study and had no radiographic signs of OA in knee or hip. Data on radiographic OA progression were available for CHECK (10 year FU), and the Rotterdam Study (6.5 year FU). Progression was defined as any increase in KL score, resulting in a KL score of ≥ 2 at follow-up. Thus, both incidence (KL score of 0 or 1 at baseline and ≥ 2 at follow-up) and progression (increase at KL score with a baseline of ≥ 2) were defined as progression in our analyses.

### Metabolite measurement

EDTA plasma samples were shipped to Nightingale to perform standardized metabolomics analyses on a high throughput platform (Nightingale Ltd, Helsinki, Finland). The 1H-NMR technique used by Nightingale provides simultaneous quantification of routine lipids, lipoprotein subclass profiling with lipid concentration units, resulting in 231 measurements. Details of the techniques have been described before^48,49^.

### Statistical analyses

Metabolites with more than 5% below detection limit were removed from analysis. Principal component analysis (PCA) was applied to reduce the data dimension of correlated metabolites. PCA groups variables (metabolites) into factors in order to explain variance present in the data. Factors with an eigenvalue above 1 after Varimax rotation with Kaiser Normalization were included in analyses. A metabolite was said to load on a given factor if its factor loading was > 0.4 or < − 0.4. For each subject, a score was computed for the measures loaded on the factor, representing the linear relationship (Pearson correlation under Varimax rotation) between a factor and variable.

The remaining factors were included in logistic regression analyses to assess their association with OA, adjusting for age, sex and BMI and multiple testing. As it is hard to interpret the overall meaning of a factor, the individual metabolites of factors with significant associations to both OA and TJA were included in individual logistic regression analyses after LN-transformation and Z-standardization. All P-values were adjusted according to Bonferroni.

Since follow-up was performed at different time points, the progression analyses were done by means of meta-analysis. The summary statistics (regression coefficients and standard errors) of CHECK were combined with the summary statistics of the Rotterdam Study in a random effects meta-analysis. To assess the modifying role of fasting, analyses were also performed without an adjustment. Analyses were performed by means of IBM SPSS Statistics version 24 and the “meta-package” of R.

## Supplementary information

Supplementary Information.
