# Supplementary material for: Serum fatty acid chain length associates with prevalent symptomatic end-stage osteoarthritis, independent of BMI
Source: Sci Rep. 2020 Sep 22;10:15459. doi: 10.1038/s41598-020-71811-3 (PMC7508826; doi:10.1038/s41598-020-71811-3)

**SUPPLEMENT**

**Serum fatty acid chain length associates with prevalent**

**symptomatic end-stage osteoarthritis, independent of BMI.**

**Running title: Metabolomics in hip & knee osteoarthritis**

J. M. T. A. Meessen^1,2^, F. Saberi-Hosnijeh^3,4^, N. Bomer^2^, W. den Hollander^2^, J.G. van der Bom^5^, J. A. van Hilten^5^, W.E. van Spil^6^, C. So-Osman^5^, A. G. Uitterlinden^3^, M. Kloppenburg^7^, R. G. H. H. Nelissen^1^, C.M. van Duijn^8,9^, P. E. Slagboom^2,8*^, J. B. J. van Meurs^3^, I. Meulenbelt^2^

**Legend:**

**Supplementary Table 1**; Distribution of cases and controls contributed by the OA cohorts of the BBMRI consortium

**Supplementary Table 2:** Individual metabolites and their respective factor load.

**Supplementary Table-3**: Total variance explained by factors identified by Principal Component Analysis.

**Supplementary Table 4**: Cross-sectional analyses of significant factors of overall OA, stratified by joint site (hip and knee)

**Supplementary Table 5**: Cross-sectional analyses of significant factors of total joint arthroplasty, stratified by joint site (hip and knee)

**Supplementary Table 6**: Impact of correction for fasting status on factors of interest for total joint arthroplasty

**Supplementary Table 7**: Impact of high or low BMI on factors of interest for total joint arthroplasty

**Supplementary Table 8**: Association of individual metabolites that load on Factor 1, 6, 17, 22 and 23 with TJA

**Supplementary Figure 1**: Fatty Acid Chain Length and OA progression

| **Supplementary Table 1**; Distribution of cases and controls contributed by the OA cohorts of the BBMRI consortium | | | | | | | | | | | |
| --- | --- | --- | --- | --- | --- | --- | --- | --- | --- | --- | --- |
|  | **Controls** | **OA + TJA** | | |  | **Arthroplasties** | | |  | **Progression** | **Controls** |
|  |  | **All** | **Hip** | **Knee** |  | **All** | **Hip** | **Knee** |  |  |  |
| CHECK | 864 | 0 | 0 | 0 |  | 0 | 0 | 0 |  | 130 | 522 |
| GARP/NORREF | 34 | 216 | 108 | 150 |  | 40 | 34 | 7 |  | - | - |
| LUMC-cohorts | 0 | 455 | 302 | 153 |  | 455 | 302 | 153 |  | - | - |
| Rotterdam Study | 1227 | 885 | 326 | 687 |  | 145 | 111 | 39 |  | 305 | 721 |
| **Total** | **2125** | **1556** | **736** | **990** |  | **640** | **447** | **199** |  | **435** | **1243** |

| Supplementary Table 2: Individual metabolites and their respective factor load. | | | | | | | | | | | | | | | | | | | | | | | | | | | | | |
| --- | --- | --- | --- | --- | --- | --- | --- | --- | --- | --- | --- | --- | --- | --- | --- | --- | --- | --- | --- | --- | --- | --- | --- | --- | --- | --- | --- | --- | --- |
| Metabolite | Abbreviation | 1 | 2 | 3 | 4 | 5 | 6 | 7 | | 8 | | 9 | 10 | 11 | 12 | 13 | 14 | 15 | 16 | 17 | 18 | 19 | | 20 | | 21 | 22 | | 23 |
| AcetoAcetate | AcAce |  |  |  |  |  |  |  | |  | |  |  |  |  |  |  |  |  |  |  |  | |  | |  |  | | 0.63 |
| Acetate | Ace |  |  |  |  |  |  |  | |  | |  |  |  |  |  |  |  |  |  |  |  | |  | |  |  | | 0.77 |
| Alanine | Ala |  |  |  |  |  |  |  | |  | |  |  |  |  |  |  |  |  | 0.62 |  |  | |  | |  |  | |  |
| Albumine | Alb |  |  |  |  |  |  |  | |  | |  |  |  |  |  |  |  |  |  | 0.69 |  | |  | |  |  | |  |
| 3-hydroxybutyrate | bOHBut |  |  |  |  |  |  |  | |  | |  |  |  |  |  |  |  |  |  |  |  | | 0.59 | |  |  | | 0.41 |
| Citrate | Cit |  |  |  |  |  |  |  | |  | |  |  |  |  |  |  |  |  |  |  |  | | 0.60 | |  |  | |  |
| Creatinine | Crea |  |  |  |  |  |  |  | |  | |  |  |  |  |  |  |  |  |  |  |  | |  | |  |  | |  |
| Glucose | Glc |  |  |  |  |  |  |  | |  | |  |  |  |  |  |  |  |  |  |  |  | |  | |  |  | |  |
| Glutamine | Gln |  |  |  |  |  |  |  | |  | |  |  |  |  |  |  |  |  |  |  | 0.77 | |  | |  |  | |  |
| Glycoprotein | Gp |  | 0.58 |  |  |  |  |  | |  | |  |  |  |  |  |  |  |  |  |  |  | |  | |  |  | |  |
| Histidine | His |  |  |  |  |  |  |  | |  | |  |  |  |  |  |  |  |  |  |  | 0.67 | |  | |  |  | |  |
| Isoleucine | Ile |  | 0.50 |  |  |  |  |  | | 0.73 | |  |  |  |  |  |  |  |  |  |  |  | |  | |  |  | |  |
| Lactate | Lac |  |  |  |  |  |  |  | |  | |  |  |  |  |  |  |  |  | 0.82 |  |  | |  | |  |  | |  |
| Leucine | Leu |  |  |  |  |  |  |  | | 0.86 | |  |  |  |  |  |  |  |  |  |  |  | |  | |  |  | |  |
| Phenylalanine | Phe |  |  |  |  |  |  |  | | 0.70 | |  |  |  |  |  |  |  |  |  |  |  | |  | |  |  | |  |
| Pyruvate | Pyr |  |  |  |  |  |  |  | |  | |  |  |  |  |  |  |  |  | 0.81 |  |  | |  | |  |  | |  |
| Tyrosine | Tyr |  |  |  |  |  |  |  | | 0.71 | |  |  |  |  |  |  |  |  |  |  |  | |  | |  |  | |  |
| Valine | Val |  |  |  |  |  |  |  | | 0.83 | |  |  |  |  |  |  |  |  |  |  |  | |  | |  |  | |  |
| Metabolite | Abbreviation | 1 | 2 | 3 | 4 | 5 | 6 | 7 | | 8 | | 9 | 10 | 11 | 12 | 13 | 14 | 15 | 16 | 17 | 18 | 19 | | 20 | | 21 | 22 | | 23 |
| Esterified Cholesterol | EstC | 0.91 |  |  |  |  |  |  | |  | |  |  |  |  |  |  |  |  |  |  |  | |  | |  |  | |  |
| Free Cholesterol | FreeC | 0.89 |  |  |  |  |  |  | |  | |  |  |  |  |  |  |  |  |  |  |  | |  | |  |  | |  |
| HDL2-C hdl2 cholesterol particle density | HDL2C |  | -0.43 | 0.80 |  |  |  |  | |  | |  |  |  |  |  |  |  |  |  |  |  | |  | |  |  | |  |
| HDL3-C hdl3 cholesterol particle density | HDL3C | 0.73 |  |  |  |  |  |  | |  | |  |  |  |  |  |  |  |  |  |  |  | |  | |  |  | |  |
| HDL-C hdl cholesterol | HDLC |  |  | 0.79 |  |  |  |  | |  | |  |  |  |  |  |  |  |  |  |  |  | |  | |  |  | |  |
| Rem t-C non-hdl / ldl cholesterol | RemtC | 0.79 | 0.58 |  |  |  |  |  | |  | |  |  |  |  |  |  |  |  |  |  |  | |  | |  |  | |  |
| Serum-C cholesterol | SerumC | 0.91 |  |  |  |  |  |  | |  | |  |  |  |  |  |  |  |  |  |  |  | |  | |  |  | |  |
| Triglycerides | SerumTG |  | 0.90 |  |  |  |  |  | |  | |  |  |  |  |  |  |  |  |  |  |  | |  | |  |  | |  |
| Metabolite | Abbreviation | 1 | 2 | 3 | 4 | 5 | 6 | 7 | 8 | | 9 | | 10 | 11 | 12 | 13 | 14 | 15 | 16 | 17 | 18 | 19 | 20 | | 21 | | 22 | 23 | |
| Apolipoprotein A-I | ApoA1 |  |  | 0.80 |  | 0.41 |  |  | |  | |  |  |  |  |  |  |  |  |  |  |  | |  | |  |  | |  |
| Apolipoprotein B | ApoB | 0.77 | 0.60 |  |  |  |  |  | |  | |  |  |  |  |  |  |  |  |  |  |  | |  | |  |  | |  |
| ratio ApoB / ApoA1 | ApoB ApoA1 | 0.56 | 0.62 | -0.48 |  |  |  |  | |  | |  |  |  |  |  |  |  |  |  |  |  | |  | |  |  | |  |
| Docosahexaenoic acid 22:6 | DHA |  |  |  |  |  |  | 0.86 | |  | |  |  |  |  |  |  |  |  |  |  |  | |  | |  |  | |  |
| DHA / total fatty acids ratio | DHA FA |  |  |  |  |  |  | 0.94 | |  | |  |  |  |  |  |  |  |  |  |  |  | |  | |  |  | |  |
| Estimated fatty chain length | Falen | -0.52 |  |  |  |  |  |  | |  | |  |  |  |  |  |  |  |  |  |  |  | |  | |  | 0.46 | |  |
| omega 3 fatty acid | FAw3 |  |  |  |  |  |  | 0.81 | |  | |  |  |  |  |  |  |  |  |  |  |  | |  | |  |  | |  |
| FAw3 / total fatty acids ratio | FAw3 fa |  |  |  |  |  |  | 0.94 | |  | |  |  |  |  |  |  |  |  |  |  |  | |  | |  |  | |  |
| omega 6 fatty acid | FAw6 | 0.74 |  |  |  |  |  |  | |  | |  |  | 0.46 |  |  |  |  |  |  |  |  | |  | |  |  | |  |
| FAw6 / total fatty acids ratio | FAw6 FA |  | -0.59 |  |  |  |  |  | |  | |  |  | 0.61 |  |  |  |  |  |  |  |  | |  | |  |  | |  |
| Linoleic acid 18:2 | LA | 0.69 |  |  |  |  |  |  | |  | |  |  | 0.55 |  |  |  |  |  |  |  |  | |  | |  |  | |  |
| LA / total fatty acids ratio | LA FA |  | -0.42 |  |  |  |  |  | |  | |  |  | 0.67 |  |  |  |  |  |  |  |  | |  | |  |  | |  |
| Monounsaturated fatty acids 16:1 18:1 | MUFA |  | 0.80 |  |  |  |  |  | |  | |  |  |  |  |  |  |  |  |  |  |  | |  | |  |  | |  |
| MUFA / total fatty acids ratio | MUFAFA |  | 0.60 |  |  |  |  |  | |  | |  |  | -0.58 |  |  |  |  |  |  |  |  | |  | |  |  | |  |
| Phosphatidycholine and other cholines | PC | 0.52 |  | 0.59 |  |  |  |  | |  | |  |  |  |  |  |  |  |  |  |  |  | |  | |  |  | |  |
| polyunsaturated fatty acids | PUFA | 0.74 |  |  |  |  |  |  | |  | |  |  | 0.42 |  |  |  |  |  |  |  |  | |  | |  |  | |  |
| PUFA / total fatty acids ratio | PUFA FA |  | -0.62 |  |  |  |  |  | |  | |  |  | 0.58 |  |  |  |  |  |  |  |  | |  | |  |  | |  |
| Saturated fatty acids | SFA | 0.48 | 0.70 |  |  |  |  |  | |  | |  |  |  |  |  |  |  |  |  |  |  | |  | |  |  | |  |
| SFA / total fatty acids ratio | SFA FA |  |  |  |  |  |  |  | |  | |  |  |  |  |  |  |  |  |  |  |  | |  | |  | -0.83 | |  |
| sphingomyelines | SM | 0.71 |  |  |  |  |  |  | |  | |  |  |  |  |  |  |  |  |  |  |  | |  | |  |  | |  |
| ratio triglycerides / phosphoglycerides | TG PG |  | 0.81 | -0.40 |  |  |  |  | |  | |  |  |  |  |  |  |  |  |  |  |  | |  | |  |  | |  |
| cholines | TotCho | 0.63 |  | 0.58 |  |  |  |  | |  | |  |  |  |  |  |  |  |  |  |  |  | |  | |  |  | |  |
| total fatty acids | TotFA | 0.56 | 0.69 |  |  |  |  |  | |  | |  |  |  |  |  |  |  |  |  |  |  | |  | |  |  | |  |
| phosphoglycerides | TotPG | 0.55 |  | 0.56 |  |  |  |  | |  | |  |  |  |  |  |  |  |  |  |  |  | |  | |  |  | |  |
| estimated degree of unsaturaization | UnsatDeg |  | -0.54 |  |  |  |  | 0.45 | |  | |  |  |  |  |  |  |  |  |  |  |  | |  | |  | 0.44 | |  |

| Continued Supplementary Table 2: Individual metabolites and their respective factor load. | | | | | | | | | | | | | | | | | | | | | | | | | | | | | | |
| --- | --- | --- | --- | --- | --- | --- | --- | --- | --- | --- | --- | --- | --- | --- | --- | --- | --- | --- | --- | --- | --- | --- | --- | --- | --- | --- | --- | --- | --- | --- |
| Metabolite | Abbreviation | 1 | 2 | 3 | | 4 | 5 | 6 | 7 | 8 | 9 | 10 | 11 | 12 | | 13 | 14 | 15 | | 16 | | 17 | | 18 | | 19 | 20 | 21 | 22 | 23 |
| HDL- mean diameter hdl particle | HDL-D |  | -0.43 | 0.87 | |  |  |  |  |  |  |  |  |  |  | |  |  | |  | |  | |  | |  |  |  |  |  |
| HDL-TG Triglycerides | HDL-TG |  | 0.74 |  | | 0.42 |  |  |  |  |  |  |  |  |  | |  |  | |  | |  | |  | |  |  |  |  |  |
| XL-HDL-C Total Cholesterol | XL HDL C |  |  | 0.81 | |  |  |  |  |  |  |  |  |  |  | |  |  | |  | |  | |  | |  |  |  |  |  |
| XL-HDL-C_% Total Choleseterol to total Lipids ratio | XL HDL C % |  |  | -0.67 | |  |  |  |  |  |  |  |  |  |  | |  | 0.43 | |  | |  | |  | |  |  |  |  |  |
| XL-HDL-CE Cholesterol Esters | XL HDL CE |  |  | 0.78 | |  |  |  |  |  |  |  |  |  |  | |  |  | |  | |  | |  | |  |  |  |  |  |
| XL-HDL-CE_% CholesterolEsters to total Lipids ratio | XL HDL CE % |  |  | -0.72 | |  |  |  |  |  |  |  |  |  |  | |  |  | |  | |  | |  | |  |  |  |  |  |
| XL-HDL-FC Free Cholesterol | XL HDL FC |  |  | 0.85 | |  |  |  |  |  |  |  |  |  |  | |  |  | |  | |  | |  | |  |  |  |  |  |
| XL-HDL-FC_% Free Cholesterol to total lipids ratio | XL HDL FC % |  |  |  | |  |  |  |  |  |  | -0.58 |  |  |  | |  |  | |  | |  | |  | |  |  |  |  |  |
| XL-HDL-L Total lipids | XL HDLL |  |  | 0.89 | |  |  |  |  |  |  |  |  |  |  | |  |  | |  | |  | |  | |  |  |  |  |  |
| XL-HDL-Particle concentration | XL HDL P |  |  | 0.89 | |  |  |  |  |  |  |  |  |  |  | |  |  | |  | |  | |  | |  |  |  |  |  |
| XL-HDL-PL Phospholipids | XL HDL PL |  | -0.44 | 0.85 | |  |  |  |  |  |  |  |  |  |  | |  |  | |  | |  | |  | |  |  |  |  |  |
| XL-HDL-PL_% Phospholipids to total Lipids ratio | XL HDL PL % |  | -0.47 | 0.64 | |  |  |  |  |  |  |  |  |  |  | |  |  | |  | |  | |  | |  |  |  |  |  |
| XL-HDL-TG Triglycerids | XL HDL TG |  | 0.57 | 0.50 | |  |  |  |  |  |  |  |  |  |  | |  |  | |  | |  | |  | |  |  |  |  |  |
| XL-HDL-TG_% TriGlycerides to total Lipids ratio | XL HDL TG % |  | 0.78 |  | |  |  |  |  |  |  |  |  |  |  | |  |  | |  | |  | |  | |  |  |  |  |  |
| L-HDL-C Total Cholesterol | L HDL C |  | -0.49 | 0.80 |  | |  |  |  |  |  |  |  |  |  | |  |  | |  | |  | | |  |  |  |  |  |  |
| L-HDL-C_% Total Choleseterol to total Lipids ratio | L HDL C % |  | -0.42 |  |  | |  |  |  |  | 0.78 |  |  |  |  | |  |  | |  | |  | | |  |  |  |  |  |  |
| L-HDL-CE Cholesterol Esters | L HDL CE |  | -0.48 | 0.79 |  | |  |  |  |  |  |  |  |  |  | |  |  | |  | |  | | |  |  |  |  |  |  |
| L-HDL-CE_% CholesterolEsters to total Lipids ratio | L HDL CE % |  |  |  |  | |  |  |  |  | 0.81 |  |  |  |  | |  |  | |  | |  | | |  |  |  |  |  |  |
| L-HDL-FC Free Cholesterol | L HDL FC |  | -0.49 | 0.78 |  | |  |  |  |  |  |  |  |  |  | |  |  | |  | |  | | |  |  |  |  |  |  |
| L-HDL-FC_% Free Cholesterol to total lipids ratio | L HDL FC % |  | -0.45 | 0.50 |  | |  |  |  |  | 0.48 |  |  |  |  | |  |  | |  | |  | | |  |  |  |  |  |  |
| L-HDL-L Total lipids | L HDLL |  | -0.46 | 0.83 |  | |  |  |  |  |  |  |  |  |  | |  |  | |  | |  | | |  |  |  |  |  |  |
| L-HDL-Particle concentration | L HDL P |  | -0.45 | 0.83 |  | |  |  |  |  |  |  |  |  |  | |  |  | |  | |  | | |  |  |  |  |  |  |
| L-HDL-PL Phospholipids | L HDL PL |  | -0.44 | 0.81 |  | |  |  |  |  |  |  |  |  |  | |  | |  | |  | |  | |  |  |  |  |  |  |
| L-HDL-PL_% Phospholipids to total Lipids ratio | L HDL PL % |  |  |  |  | |  |  |  |  | -0.70 |  |  |  |  | |  | |  | |  | |  | |  |  |  |  |  |  |
| L-HDL-TG Triglycerids | L HDL TG |  |  | 0.58 |  | |  |  |  |  |  |  |  |  |  | |  | |  | | 0.42 | |  | |  |  |  |  |  |  |
| L-HDL-TG_% TriGlycerides to total Lipids ratio | L HDL TG % |  | 0.59 |  |  | |  |  |  |  |  |  |  |  |  | |  | |  | |  | |  | |  |  |  |  |  |  |
| M-HDL-C Total Cholesterol | M HDL C |  |  | 0.60 |  | | 0.75 |  |  |  |  |  |  |  |  | |  | |  | |  | |  | |  |  |  |  |  |  |
| M-HDL-C_% Total Choleseterol to total Lipids ratio | M HDL C % |  | -0.52 |  |  | |  |  |  |  |  |  |  |  | -0.72 | |  | |  | |  | |  | |  |  |  |  |  |  |
| M-HDL-CE Cholesterol Esters | M HDL CE |  |  | 0.57 |  | | 0.75 |  |  |  |  |  |  |  |  | |  | |  | |  | |  | |  |  |  |  |  |  |
| M-HDL-CE_% CholesterolEsters to total Lipids ratio | M HDL CE % |  | -0.45 |  | -0.42 | |  |  |  |  |  |  |  |  | -0.65 | |  | |  | |  | |  | |  |  |  |  |  |  |
| M-HDL-FC Free Cholesterol | M HDL FC |  |  | 0.59 |  | | 0.64 |  |  |  |  |  |  |  |  | |  | |  | |  | |  | |  |  |  |  |  |  |
| M-HDL-FC_% Free Cholesterol to total lipids ratio | M HDL FC % | 0.41 |  |  |  | |  |  |  |  |  |  |  |  |  | |  | |  | |  | |  | |  |  |  |  |  |  |
| M-HDL-L Total lipids | M HDLL |  |  | 0.58 |  | | 0.79 |  |  |  |  |  |  |  |  | |  | |  | |  | |  | |  |  |  |  |  |  |
| M-HDL-Particle concentration mol / L | M HDL P |  |  | 0.57 |  | | 0.79 |  |  |  |  |  |  |  |  | |  | |  | |  | |  | |  |  |  |  |  |  |
| M-HDL-PL Phospholipids | M HDL PL |  |  | 0.58 |  | | 0.77 |  |  |  |  |  |  |  |  | |  | |  | |  | |  | |  |  |  |  |  |  |
| M-HDL-PL_% Phospholipids to total Lipids ratio | M HDL PL % |  |  |  |  | |  |  |  |  |  |  |  |  | 0.91 | |  | |  | |  | |  | |  |  |  |  |  |  |
| M-HDL-TG Triglycerids | M HDL TG |  | 0.76 |  |  | |  |  |  |  |  |  |  |  |  | |  | |  | |  | |  | |  |  |  |  |  |  |
| M-HDL-TG_% TriGlycerides to total Lipids ratio | M HDL TG % |  | 0.77 |  |  | |  |  |  |  |  |  |  |  |  | |  | |  | |  | |  | |  |  |  |  |  |  |
| S-HDL-C Total Cholesterol | S HDL C | 0.62 |  |  |  | | 0.44 |  |  |  |  |  |  |  |  | |  | |  | |  | |  | |  |  |  |  |  |  |
| S-HDL-C_% Total Choleseterol to total Lipids ratio | S HDL C % | 0.68 |  |  |  | |  |  |  |  |  |  |  |  |  | |  | |  | |  | |  | |  |  |  |  |  |  |
| S-HDL-CE Cholesterol Esters | S HDL CE | 0.68 |  |  |  | |  |  |  |  |  |  |  |  |  | |  | |  | |  | |  | |  |  |  |  |  |  |
| S-HDL-CE_% CholesterolEsters to total Lipids ratio | S HDL CE % | 0.69 |  |  |  | |  |  |  |  |  |  |  |  |  | |  | |  | |  | |  | |  |  |  |  |  |  |
| S-HDL-FC Free Cholesterol | S HDL FC |  |  |  |  | | 0.80 |  |  |  |  |  |  |  |  | |  | |  | |  | |  | |  |  |  |  |  |  |
| S-HDL-FC_% Free Cholesterol to total lipids ratio | S HDL FC % |  |  |  |  | |  |  |  |  |  |  |  |  |  | |  | | 0.50 | |  | |  | |  |  |  |  |  |  |
| S-HDL-L Total lipids | S HDLL |  |  |  |  | | 0.91 |  |  |  |  |  |  |  |  | |  | |  | |  | |  | |  |  |  |  |  |  |
| S-HDL-Particle concentration mol / L | S HDL P |  |  |  |  | | 0.89 |  |  |  |  |  |  |  |  | |  | |  | |  | |  | |  |  |  |  |  |  |
| S-HDL-PL Phospholipids | S HDL PL | -0.43 |  |  |  | | 0.78 |  |  |  |  |  |  |  |  | |  | |  | |  | |  | |  |  |  |  |  |  |
| S-HDL-PL_% Phospholipids to total Lipids ratio | S HDL PL % | -0.69 |  |  |  | |  |  |  |  |  |  |  |  |  | |  | |  | |  | |  | |  |  |  |  |  |  |
| S-HDL-TG Triglycerids | S HDL TG |  | 0.73 |  | 0.49 | |  |  |  |  |  |  |  |  |  | |  | |  | |  | |  | |  |  |  |  |  |  |
| S-HDL-TG_% TriGlycerides to total Lipids ratio | S HDL TG % |  | 0.70 |  | 0.46 | |  |  |  |  |  |  |  |  |  | |  | |  | |  | |  | |  |  |  |  |  |  |

| Continued Supplementary Table 2: Individual metabolites and their respective factor load. | | | | | | | | | | | | | | | | | | | | | | | | | | | | | | | | | | | | |
| --- | --- | --- | --- | --- | --- | --- | --- | --- | --- | --- | --- | --- | --- | --- | --- | --- | --- | --- | --- | --- | --- | --- | --- | --- | --- | --- | --- | --- | --- | --- | --- | --- | --- | --- | --- | --- |
| Metabolite | **Abbreviation** | | **1** | **2** | **3** | **4** | **5** | | **6** | **7** | **8** | | **9** | **10** | **11** | **12** | | **13** | **14** | **15** | **16** | | **17** | | **18** | | **19** | | **20** | | **21** | | **22** | | **23** | |
| IDL-C Total Cholesterol | IDL C | | 0.96 |  |  |  |  | |  |  |  | |  |  |  |  | |  |  |  |  | |  | |  | |  | |  | |  | |  | |  | |
| IDL-C_% Total Choleseterol to total Lipids ratio | IDL C % | | 0.45 |  |  | -0.83 |  | |  |  |  | |  |  |  |  | |  |  |  |  | |  | |  | |  | |  | |  | |  | |  | |
| IDL-CE Cholesterol Esters | IDL CE | | 0.94 |  |  |  |  | |  |  |  | |  |  |  |  | |  |  |  |  | |  | |  | |  | |  | |  | |  | |  | |
| IDL-CE_% CholesterolEsters to total Lipids ratio | IDL CE % | |  |  |  | -0.81 |  | |  |  |  | |  |  |  |  | |  |  |  |  | |  | |  | |  | |  | |  | |  | |  | |
| IDL-FC Free Cholesterol | IDL FC | | 0.96 |  |  |  |  | |  |  |  | |  |  |  |  | |  |  |  |  | |  | |  | |  | |  | |  | |  | |  | |
| IDL-FC_% Free Cholesterol to total lipids ratio | IDL FC % | | 0.56 | -0.54 |  |  |  | |  |  |  | |  |  |  |  | |  |  |  |  | |  | |  | |  | |  | |  | |  | |  | |
| IDL-L Total lipids | IDLL | | 0.96 |  |  |  |  | |  |  |  | |  |  |  |  | |  |  |  |  | |  | |  | |  | |  | |  | |  | |  | |
| IDL-Particle concentration mol / L | IDL P | | 0.96 |  |  |  |  | |  |  |  | |  |  |  |  | |  |  |  |  | |  | |  | |  | |  | |  | |  | |  | |
| IDL-PL Phospholipids | IDL PL | | 0.98 |  |  |  |  | |  |  |  | |  |  |  |  | |  |  |  |  | |  | |  | |  | |  | |  | |  | |  | |
| IDL-PL_% Phospholipids to total Lipids ratio | IDL PL % | |  | -0.78 |  |  |  | |  |  |  | |  |  |  |  | |  |  |  |  | |  | |  | |  | |  | |  | |  | |  | |
| IDL-TG Triglycerids | IDL TG | | 0.44 | 0.43 |  | 0.76 |  | |  |  |  | |  |  |  |  | |  |  |  |  | |  | |  | |  | |  | |  | |  | |  | |
| IDL-TG_% TriGlycerides to total Lipids ratio | IDL TG % | | -0.41 |  |  | 0.81 |  | |  |  |  | |  |  |  |  | |  |  |  |  | |  | |  | |  | |  | |  | |  | |  | |
| Metabolite | | Abbreviation | 1 | 2 | 3 | 4 | | 5 | 6 | 7 | | 8 | 9 | 10 | 11 | | 12 | 13 | 14 | 15 | | 16 | | 17 | | 18 | | 19 | | 20 | | 21 | | 22 | | 23 |
| LDL-C ldl cholestrol | | LDL C | 0.99 |  |  |  | |  |  |  | |  |  |  |  | |  |  |  |  | |  | |  | |  | |  | |  | |  | |  | |  |
| LDL- mean diameter ldl particle | | LDL D |  |  |  |  | |  |  |  | |  |  | 0.63 |  | |  |  |  |  | |  | |  | |  | |  | |  | |  | |  | |  |
| LDL-TG triglycerides | | LDL TG | 0.48 |  |  | 0.77 | |  |  |  | |  |  |  |  | |  |  |  |  | |  | |  | |  | |  | |  | |  | |  | |  |
| L-LDL-C Total Cholesterol | | LLDL C | 0.98 |  |  |  | |  |  |  | |  |  |  |  | |  |  |  |  | |  | |  | |  | |  | |  | |  | |  | |  |
| L-LDL-C_% Total Choleseterol to total Lipids ratio | | LLDL C % | 0.79 |  |  | -0.55 | |  |  |  | |  |  |  |  | |  |  |  |  | |  | |  | |  | |  | |  | |  | |  | |  |
| L-LDL-CE Cholesterol Esters | | LLDL CE | 0.98 |  |  |  | |  |  |  | |  |  |  |  | |  |  |  |  | |  | |  | |  | |  | |  | |  | |  | |  |
| L-LDL-CE_% CholesterolEsters to total Lipids ratio | | LLDL CE % | 0.87 |  |  |  | |  |  |  | |  |  |  |  | |  |  |  |  | |  | |  | |  | |  | |  | |  | |  | |  |
| L-LDL-FC Free Cholesterol | | LLDL FC | 0.97 |  |  |  | |  |  |  | |  |  |  |  | |  |  |  |  | |  | |  | |  | |  | |  | |  | |  | |  |
| L-LDL-FC_% Free Cholesterol to total lipids ratio | | LLDL FC % |  | -0.73 |  | -0.47 | |  |  |  | |  |  |  |  | |  |  |  |  | |  | |  | |  | |  | |  | |  | |  | |  |
| L-LDL-L Total lipids | | LLDLL | 0.98 |  |  |  | |  |  |  | |  |  |  |  | |  |  |  |  | |  | |  | |  | |  | |  | |  | |  | |  |
| L-LDL-Particle concentration mol / L | | LLDL P | 0.97 |  |  |  | |  |  |  | |  |  |  |  | |  |  |  |  | |  | |  | |  | |  | |  | |  | |  | |  |
| L-LDL-PL Phospholipids | | LLDL PL | 0.97 |  |  |  | |  |  |  | |  |  |  |  | |  |  |  |  | |  | |  | |  | |  | |  | |  | |  | |  |
| L-LDL-PL_% Phospholipids to total Lipids ratio | | LLDL PL % | -0.95 |  |  |  | |  |  |  | |  |  |  |  | |  |  |  |  | |  | |  | |  | |  | |  | |  | |  | |  |
| L-LDL-TG Triglycerids | | LLDL TG | 0.52 |  |  | 0.77 | |  |  |  | |  |  |  |  | |  |  |  |  | |  | |  | |  | |  | |  | |  | |  | |  |
| L-LDL-TG_% TriGlycerides to total Lipids ratio | | LLDL TG % | -0.41 |  |  | 0.86 | |  |  |  | |  |  |  |  | |  |  |  |  | |  | |  | |  | |  | |  | |  | |  | |  |
| M-LDL-C Total Cholesterol | | MLDL C | 0.99 |  |  |  | |  |  |  | |  |  |  |  | |  |  |  |  | |  | |  | |  | |  | |  | |  | |  | |  |
| M-LDL-C_% Total Choleseterol to total Lipids ratio | | MLDL C % | 0.83 |  |  |  | |  |  |  | |  |  |  |  | |  |  |  |  | |  | |  | |  | |  | |  | |  | |  | |  |
| M-LDL-CE Cholesterol Esters | | MLDL CE | 0.99 |  |  |  | |  |  |  | |  |  |  |  | |  |  |  |  | |  | |  | |  | |  | |  | |  | |  | |  |
| M-LDL-CE_% CholesterolEsters to total Lipids ratio | | MLDL CE % | 0.90 |  |  |  | |  |  |  | |  |  |  |  | |  |  |  |  | |  | |  | |  | |  | |  | |  | |  | |  |
| M-LDL-FC Free Cholesterol | | MLDL FC | 0.95 |  |  |  | |  |  |  | |  |  |  |  | |  |  |  |  | |  | |  | |  | |  | |  | |  | |  | |  |
| M-LDL-FC_% Free Cholesterol to total lipids ratio | | MLDL FC % | -0.85 |  |  |  | |  |  |  | |  |  |  |  | |  |  |  |  | |  | |  | |  | |  | |  | |  | |  | |  |
| M-LDL-L Total lipids | | MLDLL | 0.97 |  |  |  | |  |  |  | |  |  |  |  | |  |  |  |  | |  | |  | |  | |  | |  | |  | |  | |  |
| M-LDL-Particle concentration mol / L | | MLDL P | 0.97 |  |  |  | |  |  |  | |  |  |  |  | |  |  |  |  | |  | |  | |  | |  | |  | |  | |  | |  |
| M-LDL-PL Phospholipids | | MLDL PL | 0.89 |  |  |  | |  |  |  | |  |  |  |  | |  |  |  |  | |  | |  | |  | |  | |  | |  | |  | |  |
| M-LDL-PL_% Phospholipids to total Lipids ratio | | MLDL PL % | -0.92 |  |  |  | |  |  |  | |  |  |  |  | |  |  |  |  | |  | |  | |  | |  | |  | |  | |  | |  |
| M-LDL-TG Triglycerids | | MLDL TG | 0.47 |  |  | 0.79 | |  |  |  | |  |  |  |  | |  |  |  |  | |  | |  | |  | |  | |  | |  | |  | |  |
| M-LDL-TG_% TriGlycerides to total Lipids ratio | | MLDL TG % |  |  |  | 0.89 | |  |  |  | |  |  |  |  | |  |  |  |  | |  | |  | |  | |  | |  | |  | |  | |  |
| S-LDL-C Total Cholesterol | | SLDL C | 0.98 |  |  |  | |  |  |  | |  |  |  |  | |  |  |  |  | |  | |  | |  | |  | |  | |  | |  | |  |
| S-LDL-C_% Total Choleseterol to total Lipids ratio | | SLDL C % | 0.84 |  |  |  | |  |  |  | |  |  |  |  | |  |  |  |  | |  | |  | |  | |  | |  | |  | |  | |  |
| S-LDL-CE Cholesterol Esters | | SLDL CE | 0.99 |  |  |  | |  |  |  | |  |  |  |  | |  |  |  |  | |  | |  | |  | |  | |  | |  | |  | |  |
| S-LDL-CE_% CholesterolEsters to total Lipids ratio | | SLDL CE % | 0.90 |  |  |  | |  |  |  | |  |  |  |  | |  |  |  |  | |  | |  | |  | |  | |  | |  | |  | |  |
| S-LDL-FC Free Cholesterol | | SLDL FC | 0.90 |  |  |  | |  |  |  | |  |  |  |  | |  |  |  |  | |  | |  | |  | |  | |  | |  | |  | |  |
| S-LDL-FC_% Free Cholesterol to total lipids ratio | | SLDL FC % | -0.78 |  |  |  | |  |  |  | |  |  |  |  | |  |  |  |  | |  | |  | |  | |  | |  | |  | |  | |  |
| S-LDL-L Total lipids | | SLDLL | 0.95 |  |  |  | |  |  |  | |  |  |  |  | |  |  |  |  | |  | |  | |  | |  | |  | |  | |  | |  |
| S-LDL-Particle concentration mol / L | | SLDL P | 0.94 |  |  |  | |  |  |  | |  |  |  |  | |  |  |  |  | |  | |  | |  | |  | |  | |  | |  | |  |
| S-LDL-PL Phospholipids | | SLDL PL | 0.82 |  |  |  | |  |  |  | |  |  |  |  | |  |  |  |  | |  | |  | |  | |  | |  | |  | |  | |  |
| S-LDL-PL_% Phospholipids to total Lipids ratio | | SLDL PL % | -0.94 |  |  |  | |  |  |  | |  |  |  |  | |  |  |  |  | |  | |  | |  | |  | |  | |  | |  | |  |
| S-LDL-TG Triglycerids | | SLDL TG |  | 0.57 |  | 0.69 | |  |  |  | |  |  |  |  | |  |  |  |  | |  | |  | |  | |  | |  | |  | |  | |  |
| S-LDL-TG_% TriGlycerides to total Lipids ratio | | SLDL TG % |  | 0.49 |  | 0.76 | |  |  |  | |  |  |  |  | |  |  |  |  | |  | |  | |  | |  | |  | |  | |  | |  |

| Continued Supplementary Table 2: Individual metabolites and their respective factor load. | | | | | | | | | | | | | | | | | | | | | | | | |
| --- | --- | --- | --- | --- | --- | --- | --- | --- | --- | --- | --- | --- | --- | --- | --- | --- | --- | --- | --- | --- | --- | --- | --- | --- |
| Metabolite | Abbreviation | 1 | 2 | 3 | 4 | 5 | 6 | 7 | 8 | 9 | 10 | 11 | 12 | 13 | 14 | 15 | 16 | 17 | 18 | 19 | 20 | 21 | 22 | 23 |
| VLDL-C - vldl cholesterol | VLDL C | 0.55 | 0.80 |  |  |  |  |  |  |  |  |  |  |  |  |  |  |  |  |  |  |  |  |  |
| VLDL- mean diameter vldl particles | VLDL D |  | 0.90 |  |  |  |  |  |  |  |  |  |  |  |  |  |  |  |  |  |  |  |  |  |
| VLDL-Triglycerides | VLDL TG |  | 0.91 |  |  |  |  |  |  |  |  |  |  |  |  |  |  |  |  |  |  |  |  |  |
| XXL-VLDL-C Total Cholesterol | XXLVLDL C |  | 0.93 |  |  |  |  |  |  |  |  |  |  |  |  |  |  |  |  |  |  |  |  |  |
| XXL-VLDL-C_% Total Choleseterol to total Lipids ratio | XXLVLDL C % |  |  |  |  |  | 0.54 |  |  |  |  |  | 0.68 |  |  |  |  |  |  |  |  |  |  |  |
| XXL-VLDL-CE Cholesterol Esters | XXLVLDL CE |  | 0.87 |  |  |  |  |  |  |  |  |  |  |  |  |  |  |  |  |  |  |  |  |  |
| XXL-VLDL-CE_% CholesterolEsters to total Lipids ratio | XXLVLDL CE % |  |  |  |  |  | 0.62 |  |  |  |  |  | 0.44 |  |  |  |  |  |  |  |  |  |  |  |
| XXL-VLDL-FC Free Cholesterol | XXLVLDL FC |  | 0.95 |  |  |  |  |  |  |  |  |  |  |  |  |  |  |  |  |  |  |  |  |  |
| XXL-VLDL-FC_% Free Cholesterol to total lipids ratio | XXLVLDL FC % |  |  |  |  |  |  |  |  |  |  |  | 0.68 |  |  |  |  |  |  |  |  |  |  |  |
| XXL-VLDL-L Total lipids | XXLVLDLL |  | 0.93 |  |  |  |  |  |  |  |  |  |  |  |  |  |  |  |  |  |  |  |  |  |
| XXL-VLDL-Particle concentration mol / L | XXLVLDL P |  | 0.93 |  |  |  |  |  |  |  |  |  |  |  |  |  |  |  |  |  |  |  |  |  |
| XXL-VLDL-PL Phospholipids | XXLVLDL PL |  | 0.92 |  |  |  |  |  |  |  |  |  |  |  |  |  |  |  |  |  |  |  |  |  |
| XXL-VLDL-PL_% Phospholipids to total Lipids ratio | XXLVLDL PL % |  |  |  |  |  |  |  |  |  |  |  | 0.44 |  |  |  |  |  |  |  |  |  |  |  |
| XXL-VLDL-TG Triglycerids | XXLVLDL TG |  | 0.92 |  |  |  |  |  |  |  |  |  |  |  |  |  |  |  |  |  |  |  |  |  |
| XXL-VLDL-TG_% TriGlycerides to total Lipids ratio | XXLVLDL TG % |  |  |  |  |  |  |  |  |  |  |  | -0.81 |  |  |  |  |  |  |  |  |  |  |  |
| XL-VLDL-C Total Cholesterol | XLVLDL C |  | 0.96 |  |  |  |  |  |  |  |  |  |  |  |  |  |  |  |  |  |  |  |  |  |
| XL-VLDL-C_% Total Choleseterol to total Lipids ratio | XLVLDL C % |  | -0.54 |  |  |  | 0.73 |  |  |  |  |  |  |  |  |  |  |  |  |  |  |  |  |  |
| XL-VLDL-CE Cholesterol Esters | XLVLDL CE |  | 0.95 |  |  |  |  |  |  |  |  |  |  |  |  |  |  |  |  |  |  |  |  |  |
| XL-VLDL-CE_% CholesterolEsters to total Lipids ratio | XLVLDL CE % |  | -0.46 |  |  |  | 0.71 |  |  |  |  |  |  |  |  |  |  |  |  |  |  |  |  |  |
| XL-VLDL-FC Free Cholesterol | XLVLDL FC |  | 0.94 |  |  |  |  |  |  |  |  |  |  |  |  |  |  |  |  |  |  |  |  |  |
| XL-VLDL-FC_% Free Cholesterol to total lipids ratio | XLVLDL FC % |  | -0.46 |  |  |  | 0.61 |  |  |  |  |  |  |  |  |  |  |  |  |  |  |  |  |  |
| XL-VLDL-L Total lipids | XLVLDLL |  | 0.96 |  |  |  |  |  |  |  |  |  |  |  |  |  |  |  |  |  |  |  |  |  |
| XL-VLDL-Particle concentration mol / L | XLVLDL P |  | 0.96 |  |  |  |  |  |  |  |  |  |  |  |  |  |  |  |  |  |  |  |  |  |
| XL-VLDL-PL Phospholipids | XLVLDL PL |  | 0.93 |  |  |  |  |  |  |  |  |  |  |  |  |  |  |  |  |  |  |  |  |  |
| XL-VLDL-PL_% Phospholipids to total Lipids ratio | XLVLDL PL % |  |  |  |  |  |  |  |  |  |  |  |  |  | 0.46 |  |  |  |  |  |  |  |  |  |
| XL-VLDL-TG Triglycerids | XLVLDL TG |  | 0.93 |  |  |  |  |  |  |  |  |  |  |  |  |  |  |  |  |  |  |  |  |  |
| XL VLDL TG % | XLVLDL TG % |  |  |  |  |  | -0.68 |  |  |  |  |  |  |  |  |  |  |  |  |  |  |  |  |  |
| L-VLDL-C Total Cholesterol | LVLDL C |  | 0.96 |  |  |  |  |  |  |  |  |  |  |  |  |  |  |  |  |  |  |  |  |  |
| L-VLDL-C_% Total Choleseterol to total Lipids ratio | LVLDL C % |  |  |  |  |  | 0.69 |  |  |  |  |  |  |  |  |  |  |  |  |  |  |  |  |  |
| L-VLDL-CE Cholesterol Esters | LVLDL CE |  | 0.95 |  |  |  |  |  |  |  |  |  |  |  |  |  |  |  |  |  |  |  |  |  |
| L-VLDL-CE_% CholesterolEsters to total Lipids ratio | LVLDL CE % |  |  |  |  |  | 0.72 |  |  |  |  |  |  |  |  |  |  |  |  |  |  |  |  |  |
| L-VLDL-FC Free Cholesterol | LVLDL FC |  | 0.94 |  |  |  |  |  |  |  |  |  |  |  |  |  |  |  |  |  |  |  |  |  |
| L-VLDL-FC_% Free Cholesterol to total lipids ratio | LVLDL FC % |  |  |  |  |  |  |  |  |  |  |  |  |  | 0.69 |  |  |  |  |  |  |  |  |  |
| L-VLDL-L Total lipids | LVLDLL |  | 0.94 |  |  |  |  |  |  |  |  |  |  |  |  |  |  |  |  |  |  |  |  |  |
| L-VLDL-Particle concentration mol / L | LVLDL P |  | 0.93 |  |  |  |  |  |  |  |  |  |  |  |  |  |  |  |  |  |  |  |  |  |
| L-VLDL-PL Phospholipids | LVLDL PL |  | 0.92 |  |  |  |  |  |  |  |  |  |  |  |  |  |  |  |  |  |  |  |  |  |
| L-VLDL-PL_% Phospholipids to total Lipids ratio | LVLDL PL % |  | 0.41 |  |  |  |  |  |  |  |  |  |  |  |  |  |  |  |  |  |  | 0.63 |  |  |
| L-VLDL-TG Triglycerids | LVLDL TG |  | 0.91 |  |  |  |  |  |  |  |  |  |  |  |  |  |  |  |  |  |  |  |  |  |
| L-VLDL-TG_% TriGlycerides to total Lipids ratio | LVLDL TG % |  |  |  |  |  | -0.52 |  |  |  |  |  |  |  | -0.47 |  |  |  |  |  |  |  |  |  |
| M-VLDL-C Total Cholesterol | MVLDL C |  | 0.91 |  |  |  |  |  |  |  |  |  |  |  |  |  |  |  |  |  |  |  |  |  |
| M-VLDL-C_% Total Choleseterol to total Lipids ratio | MVLDL C % | 0.49 |  |  |  |  | 0.55 |  |  |  |  |  |  |  |  |  |  |  |  |  |  |  |  |  |
| M-VLDL-CE Cholesterol Esters | MVLDL CE | 0.43 | 0.86 |  |  |  |  |  |  |  |  |  |  |  |  |  |  |  |  |  |  |  |  |  |
| M-VLDL-CE_% CholesterolEsters to total Lipids ratio | MVLDL CE % | 0.48 | -0.41 |  |  |  | 0.55 |  |  |  |  |  |  |  |  |  |  |  |  |  |  |  |  |  |
| M-VLDL-FC Free Cholesterol | MVLDL FC |  | 0.92 |  |  |  |  |  |  |  |  |  |  |  |  |  |  |  |  |  |  |  |  |  |
| M-VLDL-FC_% Free Cholesterol to total lipids ratio | MVLDL FC % |  |  |  |  |  |  |  |  |  |  |  |  |  | 0.45 |  |  |  |  |  |  |  |  |  |
| M-VLDL-L Total lipids | MVLDLL |  | 0.93 |  |  |  |  |  |  |  |  |  |  |  |  |  |  |  |  |  |  |  |  |  |
| M-VLDL-Particle concentration mol / L | MVLDL P |  | 0.92 |  |  |  |  |  |  |  |  |  |  |  |  |  |  |  |  |  |  |  |  |  |
| M-VLDL-PL Phospholipids | MVLDL PL |  | 0.92 |  |  |  |  |  |  |  |  |  |  |  |  |  |  |  |  |  |  |  |  |  |
| M-VLDL-PL_% Phospholipids to total Lipids ratio | MVLDL PL % |  |  |  |  |  |  |  |  |  |  |  |  |  |  |  |  |  |  |  |  | 0.75 |  |  |
| M-VLDL-TG Triglycerids | MVLDL TG |  | 0.89 |  |  |  |  |  |  |  |  |  |  |  |  |  |  |  |  |  |  |  |  |  |
| M-VLDL-TG_% TriGlycerides to total Lipids ratio | MVLDL TG % | -0.44 |  |  |  |  | -0.54 |  |  |  |  |  |  |  |  |  |  |  |  |  |  |  |  |  |

| Continued Supplementary Table 2: Individual metabolites and their respective factor load. | | | | | | | | | | | | | | | | | | | | | | | | |  |
| --- | --- | --- | --- | --- | --- | --- | --- | --- | --- | --- | --- | --- | --- | --- | --- | --- | --- | --- | --- | --- | --- | --- | --- | --- | --- |
| Metabolite | Abbreviation | 1 | 2 | 3 | 4 | 5 | 6 | 7 | 8 | 9 | 10 | 11 | 12 | 13 | 14 | 15 | 16 | 17 | 18 | 19 | 20 | 21 | 22 | 23 | |
| S-VLDL-C Total Cholesterol | SVLDL C | 0.69 | 0.65 |  |  |  |  |  |  |  |  |  |  |  |  |  |  |  |  |  |  |  |  |  | |
| S-VLDL-C_% Total Choleseterol to total Lipids ratio | SVLDL C % | 0.51 | -0.58 |  |  |  |  |  |  |  |  |  |  |  |  |  |  |  |  |  |  |  |  |  | |
| S-VLDL-CE Cholesterol Esters | SVLDL CE | 0.78 | 0.52 |  |  |  |  |  |  |  |  |  |  |  |  |  |  |  |  |  |  |  |  |  | |
| S-VLDL-CE_% CholesterolEsters to total Lipids ratio | SVLDL CE % | 0.50 | -0.56 |  |  |  |  |  |  |  |  |  |  |  |  |  |  |  |  |  |  |  |  |  | |
| S-VLDL-FC Free Cholesterol | SVLDL FC | 0.48 | 0.77 |  |  |  |  |  |  |  |  |  |  |  |  |  |  |  |  |  |  |  |  |  | |
| S-VLDL-FC_% Free Cholesterol to total lipids ratio | SVLDL FC % |  | -0.50 |  |  |  |  |  |  |  |  |  |  |  |  |  |  |  |  |  |  |  |  |  | |
| S-VLDL-L Total lipids | SVLDLL |  | 0.83 |  |  |  |  |  |  |  |  |  |  |  |  |  |  |  |  |  |  |  |  |  | |
| S-VLDL-Particle concentration mol / L | SVLDL P |  | 0.84 |  |  |  |  |  |  |  |  |  |  |  |  |  |  |  |  |  |  |  |  |  | |
| S-VLDL-PL Phospholipids | SVLDL PL |  | 0.80 |  |  |  |  |  |  |  |  |  |  |  |  |  |  |  |  |  |  |  |  |  | |
| S-VLDL-PL_% Phospholipids to total Lipids ratio | SVLDL PL % |  |  |  |  | 0.45 |  |  |  |  |  |  |  |  |  |  |  |  |  |  |  | 0.41 |  |  | |
| S-VLDL-TG Triglycerids | SVLDL TG |  | 0.85 |  |  |  |  |  |  |  |  |  |  |  |  |  |  |  |  |  |  |  |  |  | |
| S-VLDL-TG_% TriGlycerides to total Lipids ratio | SVLDL TG % | -0.41 | 0.63 |  |  |  |  |  |  |  |  |  |  |  |  |  |  |  |  |  |  |  |  |  | |
| XS-VLDL-C Total Cholesterol | XSVLDL C | 0.89 |  |  |  |  |  |  |  |  |  |  |  |  |  |  |  |  |  |  |  |  |  |  | |
| XS-VLDL-C_% Total Choleseterol to total Lipids ratio | XSVLDL C % |  | -0.60 |  | -0.62 |  |  |  |  |  |  |  |  |  |  |  |  |  |  |  |  |  |  |  | |
| XS-VLDL-CE Cholesterol Esters | XSVLDL CE | 0.85 |  |  |  |  |  |  |  |  |  |  |  |  |  |  |  |  |  |  |  |  |  |  | |
| XS-VLDL-CE_% CholesterolEsters to total Lipids ratio | XSVLDL CE % |  | -0.50 |  | -0.61 |  |  |  |  |  |  |  |  |  |  |  |  |  |  |  |  |  |  |  | |
| XS-VLDL-FC Free Cholesterol | XSVLDL FC | 0.89 |  |  |  |  |  |  |  |  |  |  |  |  |  |  |  |  |  |  |  |  |  |  | |
| XS-VLDL-FC_% Free Cholesterol to total lipids ratio | XSVLDL FC % |  | -0.48 |  |  |  |  |  |  |  |  |  |  |  |  |  |  |  |  |  |  |  |  |  | |
| XS-VLDL-L Total lipids | XSVLDLL | 0.84 | 0.42 |  |  |  |  |  |  |  |  |  |  |  |  |  |  |  |  |  |  |  |  |  | |
| XS-VLDL-Particle concentration mol / L | XSVLDL P | 0.81 | 0.47 |  |  |  |  |  |  |  |  |  |  |  |  |  |  |  |  |  |  |  |  |  | |
| XS-VLDL-PL Phospholipids | XSVLDL PL | 0.93 |  |  |  |  |  |  |  |  |  |  |  |  |  |  |  |  |  |  |  |  |  |  | |
| XS-VLDL-PL_% Phospholipids to total Lipids ratio | XSVLDL PL % | 0.79 |  |  |  |  |  |  |  |  |  |  |  |  |  |  |  |  |  |  |  |  |  |  | |
| XS-VLDL-TG Triglycerids | XSVLDL TG |  | 0.74 |  | 0.50 |  |  |  |  |  |  |  |  |  |  |  |  |  |  |  |  |  |  |  | |
| XS-VLDL-TG_% TriGlycerides to total Lipids ratio | XSVLDL TG % |  | 0.62 |  | 0.53 |  |  |  |  |  |  |  |  |  |  |  |  |  |  |  |  |  |  |  | |
| Extraction Method: Principal Component Analysis. Rotation Method: Varimax with Kaiser Normalization. a Rotation converged in 18 iterations. | | | | | | | | | | | | | | | | | | | | | | | | | |

| Supplementary Table-3: Total variance explained by factors identified by Principal Component Analysis. | | |
| --- | --- | --- |
| Factor | Eigen value | % of Variance Explained |
| 1 | 70.93 | 31.25 |
| 2 | 57.19 | 25.19 |
| 3 | 19.81 | 8.73 |
| 4 | 11.07 | 4.88 |
| 5 | 8.54 | 3.76 |
| 6 | 4.75 | 2.09 |
| 7 | 4.08 | 1.80 |
| 8 | 3.66 | 1.61 |
| 9 | 3.04 | 1.34 |
| 10 | 2.85 | 1.25 |
| 11 | 2.72 | 1.20 |
| 12 | 2.50 | 1.10 |
| 13 | 2.04 | 0.90 |
| 14 | 1.87 | 0.82 |
| 15 | 1.84 | 0.81 |
| 16 | 1.63 | 0.72 |
| 17 | 1.60 | 0.70 |
| 18 | 1.48 | 0.65 |
| 19 | 1.39 | 0.61 |
| 20 | 1.24 | 0.55 |
| 21 | 1.15 | 0.51 |
| 22 | 1.11 | 0.49 |
| 23 | 1.00 | 0.44 |
| Total | 207.49 | 91.40 |

| **Supplementary Table 4**: Cross-sectional analyses of significant factors of overall OA, stratified by joint site (hip and knee) | | | | | | | |
| --- | --- | --- | --- | --- | --- | --- | --- |
| **Factor** | **Hip OA(N=736)** | | |  | **Knee OA(N=990)** | | |
|  | **OR** | **95%CI** | **P *_adjusted_*** |  | **OR** | **95%CI** | ***P _adjusted_*** |
| 4 | 1.38 | 1.24-1.55 | 4.5x10^-8^ |  | 1.18 | 1.08-1.30 | 0.005 |
| 11 | 0.82 | 0.74-0.91 | 6.0x10^-4^ |  | 0.89 | 0.82-0.97 | 0.045 |
| 15 | 0.85 | 0.76-0.94 | 0.010 |  | 0.85 | 0.78-0.94 | 0.005 |
| 19 | 0.67 | 0.59-0.75 | 2.1x10^-10^ |  | 0.82 | 0.74-0.91 | 0.001 |
| 20 | 0.89 | 0.80–1.00 | 0.255 |  | 0.84 | 0.76-0.93 | 0.005 |
| Analyses stratified by affected joint, adjusted for age, sex and BMI.  P-value adjusted by Bonferroni | | | | | | | |

| **Supplementary Table 5**: Cross-sectional analyses of significant factors of total joint arthroplasty, stratified by joint site (hip and knee) | | | | | | | |
| --- | --- | --- | --- | --- | --- | --- | --- |
| **Factor** | **THA(N=447)** | | |  | **TKA(N=199)** | | |
|  | **OR** | **95%CI** | ***P _adjusted_*** |  | **OR** | **95%CI** | ***P _adjusted_*** |
| 1 | 0.86 | 0.75-0.99 | 0.272 |  | 0.71 | 0.58-0.88 | 0.008 |
| 4 | 1.41 | 1.20-1.65 | 1.4x10^-4^ |  | 1.39 | 1.14-1.71 | 0.008 |
| 6 | 1.20 | 1.05-1.37 | 0.056 |  | 1.19 | 1.00-1.41 | 0.448 |
| 11 | 0.75 | 0.65-0.87 | 0.001 |  | 0.81 | 0.66-0.99 | 0.304 |
| 17 | 1.34 | 1.16-1.55 | 4.8x10^-4^ |  | 1.42 | 1.17-1.73 | 0.004 |
| 19 | 0.62 | 0.52-0.72 | 2.2x10^-8^ |  | 0.76 | 0.58-0.99 | 0.336 |
| 22 | 1.44 | 1.25-1.66 | 4.8x10^-6^ |  | 1.67 | 1.35-2.08 | 2.4x10^-5^ |
| 23 | 1.36 | 1.17-1.59 | 4.6x10^-4^ |  | 1.81 | 1.47-2.24 | 2.6x10^-7^ |
| Analyses stratified by affected joint, adjusted for age, sex and BMI.  P-value adjusted by Bonferroni | | | | | | | |

| **Supplementary Table 6:** Impact of correction for fasting status on factors of interest for total joint arthroplasty | | | | | | | |  |
| --- | --- | --- | --- | --- | --- | --- | --- | --- |
| **Factor** | | **TJA**  **corrected for age, sex and BMI** | | |  | **TJA**  **corrected for age, sex, BMI and fasting** | | |
|  | | **OR** | **95%CI** | ***P _adjusted_*** |  | **OR** | **95%CI** | ***P _adjusted_*** |
| 1 | | 0.79 | 0.70-0.89 | 2.6x10^-3^ |  | 0.79 | 0.69-0.89 | 4.4x10^-4^ |
| 4 | | 1.44 | 1.26-1.64 | 1.2x10^-6^ |  | 1.36 | 1.19-1.55 | 1.9x10^-4^ |
| 6 | | 1.20 | 1.07-1.34 | 4.5x10^-2^ |  | 1.28 | 1.14-1.145 | 9.2x10^-4^ |
| 11 | | 0.78 | 0.69-0.88 | 1.7x10^-3^ |  | 0.79 | 0.70-0.89 | 0.002 |
| 17 | | 1.36 | 1.21-1.54 | 1.2x10^-5^ |  | 1.40 | 1.24-1.58 | 1.61x10^-6^ |
| 19 | | 0.64 | 0.56-0.74 | 9.6x10^-9^ |  | 0.68 | 0.58-0.78 | 1.75x10^-6^ |
| 22 | | 1.50 | 1.33-1.70 | 2.2x10^-9^ |  | 1.45 | 1.29-1.64 | 1.85x10^-8^ |
| 23 | | 1.51 | 1.33-1.72 | 9.0x10^-9^ |  | 1.43 | 1.26-1.61 | 3.65x10^-7^ |
| P-value adjusted for Bonferroni | | | | | | | | |

| **Supplementary Table 7:** Impact of high or low BMI on factors of interest for total joint arthroplasty | | | | | | | |  |
| --- | --- | --- | --- | --- | --- | --- | --- | --- |
| **Factor** | | **TJA**  **In BMI < 30 kg/m2**  **N=3433**  **corrected for age, sex** | | |  | **TJA**  **In BMI ≥30 kg/m2**  **N=968**  **corrected for age, sex** | | |
|  | | **OR** | **95%CI** | ***P _adjusted_*** |  | **OR** | **95%CI** | ***P _adjusted_*** |
| 1 | | 0.74 | 0.64-0.85 | 5.7x10^-4^ |  | 0.90 | 0.72-1.11 | NS |
| 4 | | 1.41 | 1.21-1.65 | 2.6x10^-4^ |  | 1.55 | 1.21-1.99 | 0.010 |
| 6 | | 1.12 | 0.97-1.28 | NS |  | 1.40 | 1.11-1.78 | 0.095 |
| 11 | | 0.77 | 0.67-0.89 | 0.005 |  | 0.78 | 0.59-1.03 | NS |
| 17 | | 1.37 | 1.18-1.59 | 6.3x10^-4^ |  | 1.42 | 1.15-1.77 | 0.019 |
| 19 | | 0.59 | 0.50-0.70 | 9.3x10^-9^ |  | 0.72 | 0.57-0.92 | 0.171 |
| 22 | | 1.53 | 1.32-1.77 | 4.0x10^-7^ |  | 1.50 | 1.18-1.90 | 0.019 |
| 23 | | 1.52 | 1.31-1.77 | 1.1x10^-6^ |  | 1.34 | 1.06-1.69 | 0.285 |
| P-value adjusted for Bonferroni | | | | | | | | |

| **Supplementary Table 8:** Association of individual metabolites that load on Factor 1, 6, 17, 22 and 23 with TJA | | | | | | | | | | | | | | |
| --- | --- | --- | --- | --- | --- | --- | --- | --- | --- | --- | --- | --- | --- | --- |
| **Abbreviation** | **Factor load** | | | | | | | | | | **Cross-sectional analyses of individual metabolites with TJA** | | | |
|  | **1** | **4** | **6** | **11** | **15** | **17** | **19** | **20** | **22** | **23** | **OR** | **1/OR** | **95%CI** | **P_adjusted_** |
| AcAce |  |  |  |  |  |  |  |  |  | 0,63 | 2,55 | 0,39 | 2,09-3,11 | 1.0x10^-320^ |
| Pyr |  |  |  |  |  | 0,81 |  |  |  |  | 1,93 | 0,52 | 1,72-2,16 | 1.0x10^-320^ |
| Falen | -0,52 |  |  |  |  |  |  |  | 0,46 |  | 1,83 | 0,55 | 1,64-2,05 | 1.0x10^-320^ |
| M VLDL TG % | -0,44 |  | -0,54 |  |  |  |  |  |  |  | 1,73 | 0,58 | 1,53-1,96 | 1.0x10^-320^ |
| M LDL TG % |  | 0,89 |  |  |  |  |  |  |  |  | 1,73 | 0,58 | 1,53-1,96 | 1.0x10^-320^ |
| IDL TG % | -0,41 | 0,81 |  |  |  |  |  |  |  |  | 1,61 | 0,62 | 1,43-1,81 | 1.5x10^-13^ |
| L LDL TG % | -0,41 | 0,86 |  |  |  |  |  |  |  |  | 1,59 | 0,63 | 1,42-1,79 | 2.9x10^-13^ |
| S LDL TG % |  | 0,76 |  |  |  |  |  |  |  |  | 1,59 | 0,63 | 1,41-1,80 | 3.3x10^-12^ |
| XS VLDL TG |  |  |  |  |  |  |  |  |  |  | 1,47 | 0,68 | 1,31-1,65 | 4.6x10^-9^ |
| Lac |  |  |  |  |  | 0,82 |  |  |  |  | 1,47 | 0,68 | 1,31-1,65 | 1.1x10^-8^ |
| MUFAFA |  |  |  | -0,58 |  |  |  |  |  |  | 1,46 | 0,69 | 1,30-1,65 | 1.9x10^-8^ |
| bOHBut |  |  |  |  |  |  |  | 0,59 |  | 0,41 | 1,36 | 0,74 | 1,21-1,52 | 1.3x10^-5^ |
| S VLDL TG % | -0,41 |  |  |  |  |  |  |  |  |  | 1,36 | 0,74 | 1,20-1,55 | 3.8x10^-4^ |
| S HDL TG% |  | 0,46 |  |  |  |  |  |  |  |  | 1,32 | 0,76 | 1,17-1,48 | 3.8x10^-4^ |
| S HDL TG |  | 0,49 |  |  |  |  |  |  |  |  | 1,30 | 0,77 | 1,16-1,46 | 0.001 |
| M LDL TG | 0,47 | 0,79 |  |  |  |  |  |  |  |  | 1,26 | 0,79 | 1,12-1,42 | 0.016 |
| S LDL TG |  | 0,69 |  |  |  |  |  |  |  |  | 1,24 | 0,81 | 1,11-1,40 | 0.031 |
| M LDL PL % | -0,92 |  |  |  |  |  |  |  |  |  | 1,22 | 0,82 | 1,10-1,35 | 0.021 |
| LDL TG | 0,48 | 0,77 |  |  |  |  |  |  |  |  | 1,20 | 0,83 | 1,07-1,35 | NS |
| IDL TG | 0,44 | 0,76 |  |  |  |  |  |  |  |  | 1,18 | 0,85 | 1,05-1,32 | NS |
| S LDL PL % | -0,94 |  |  |  |  |  |  |  |  |  | 1,18 | 0,85 | 1,06-1,31 | NS |
| L LDL PL % | -0,95 |  |  |  |  |  |  |  |  |  | 1,17 | 0,85 | 1,05-1,30 | NS |
| L LDL TG | 0,52 | 0,77 |  |  |  |  |  |  |  |  | 1,16 | 0,86 | 1,03-1,30 | NS |
| M LDL FC % | -0,85 |  |  |  |  |  |  |  |  |  | 1,15 | 0,87 | 1,03-1,28 | NS |
| HDL-TG |  | 0,42 |  |  |  |  |  |  |  |  | 1,15 | 0,87 | 1,02-1,29 | NS |
| XXL VLDL C % |  |  | 0,54 |  |  |  |  |  |  |  | 1,14 | 0,88 | 1,02-1,27 | NS |
| XS VLDL TG% |  |  |  |  |  |  |  |  |  |  | 1,14 | 0,88 | 1,02-1,27 | NS |
| UnsatDeg |  |  |  |  |  |  |  |  | 0,44 |  | 1,12 | 0,89 | 0,99-1,28 | NS |
| S LDL FC % | -0,78 |  |  |  |  |  |  |  |  |  | 1,11 | 0,90 | 0,99-1,25 | NS |
| XXL VLDL CE % |  |  | 0,62 |  |  |  |  |  |  |  | 1,11 | 0,90 | 0,99-1,25 | NS |
| L VLDL TG % |  |  | -0,52 |  |  |  |  |  |  |  | 1,09 | 0,92 | 0,93-1,29 | NS |
| M HDL FC % | 0,41 |  |  |  |  |  |  |  |  |  | 1,07 | 0,93 | 0,96-1,21 | NS |
| XL VLDL TG % |  |  | -0,68 |  |  |  |  |  |  |  | 1,06 | 0,94 | 0,91-1,24 | NS |
| PC | 0,52 |  |  |  |  |  |  |  |  |  | 1,05 | 0,95 | 0,92-1,20 | NS |
| XL VLDL CE % |  |  | 0,71 |  |  |  |  |  |  |  | 1,05 | 0,95 | 0,94-1,18 | NS |
| Ace |  |  |  |  |  |  |  |  |  | 0,77 | 1,04 | 0,96 | 0,93-1,16 | NS |
| Cit |  |  |  |  |  |  |  | 0,60 |  |  | 1,01 | 0,99 | 0,90-1,14 | NS |
| S HDL CE | 0,68 |  |  |  |  |  |  |  |  |  | 1,00 | 1,00 | 0,91-1,11 | NS |
| S HDL CE % | 0,69 |  |  |  |  |  |  |  |  |  | 1,00 | 1,00 | 0,90-1,12 | NS |
| S HDL C | 0,62 |  |  |  |  |  |  |  |  |  | 0,98 | 1,02 | 0,88-1,09 | NS |
| SM | 0,71 |  |  |  |  |  |  |  |  |  | 0,98 | 1,02 | 0,87-1,09 | NS |
| S HDL C % | 0,68 |  |  |  |  |  |  |  |  |  | 0,97 | 1,03 | 0,87-1,09 | NS |
| S HDL PL | -0,43 |  |  |  |  |  |  |  |  |  | 0,97 | 1,03 | 0,86-1,09 | NS |
| XL VLDL C% |  |  | 0,73 |  |  |  |  |  |  |  | 0,97 | 1,03 | 0,86-1,10 | NS |
| S HDL PL % | -0,69 |  |  |  |  |  |  |  |  |  | 0,95 | 1,05 | 0,85-1,07 | NS |
| SFA FA |  |  |  |  |  |  |  |  | -0,83 |  | 0,95 | 1,05 | 0,84-1,07 | NS |
| S VLDL FC | 0,48 |  |  |  |  |  |  |  |  |  | 0,94 | 1,06 | 0,84-1,05 | NS |
| Histidine |  |  |  |  |  |  | 0,67 |  |  |  | 0,92 | 1,09 | 0,81-1,04 | NS |
| L LDL FC % |  | -0,47 |  |  |  |  |  |  |  |  | 0,91 | 1,10 | 0,84-0,99 | NS |
| ApoB ApoA1 | 0,56 |  |  |  |  |  |  |  |  |  | 0,89 | 1,12 | 0,80-0,99 | NS |
| L VLDL C% |  |  | 0,69 |  |  |  |  |  |  |  | 0,89 | 1,12 | 0,79-1,01 | NS |
| XL VLDL FC % |  |  | 0,61 |  |  |  |  |  |  |  | 0,88 | 1,14 | 0,78-0,99 | NS |
| TotFA | 0,56 |  |  |  |  |  |  |  |  |  | 0,87 | 1,15 | 0,77-0,99 | NS |
| L VLDL CE % |  |  | 0,72 |  |  |  |  |  |  |  | 0,86 | 1,16 | 0,86-1,07 | NS |

| **Continued Supplementary Table 8:** Association of individual metabolites that load on Factor 1, 6, 17, 22 and 23 with TJA | | | | | | | | | | | | | | |
| --- | --- | --- | --- | --- | --- | --- | --- | --- | --- | --- | --- | --- | --- | --- |
| **Abbreviation** | **Factor load** | | | | | | | | | | **Cross-sectional analyses of individual metabolites with TJA** | | | |
|  | **1** | **4** | **6** | **11** | **15** | **17** | **19** | **20** | **22** | **23** | **OR** | **1/OR** | **95%CI** | **P_adjusted_** |
| S HDL FC% |  |  |  |  | 0,50 |  |  |  |  |  | 0,86 | 1,16 | 0,77-0,96 | NS |
| SFA | 0,48 |  |  |  |  |  |  |  |  |  | 0,86 | 1,16 | 0,76-0,98 | NS |
| VLDL C | 0,55 |  |  |  |  |  |  |  |  |  | 0,85 | 1,18 | 0,76-0,96 | NS |
| XL HDL C% |  |  |  |  | 0,43 |  |  |  |  |  | 0,85 | 1,18 | 0,76-0,96 | NS |
| IDL FC % | 0,56 |  |  |  |  |  |  |  |  |  | 0,84 | 1,19 | 0,75-0,94 | NS |
| XS VLDL PL % | 0,79 |  |  |  |  |  |  |  |  |  | 0,84 | 1,19 | 0,76-0,94 | NS |
| M HDL CE % |  | -0,42 |  |  |  |  |  |  |  |  | 0,83 | 1,20 | 0,74-0,92 | NS |
| ApoB | 0,77 |  |  |  |  |  |  |  |  |  | 0,83 | 1,20 | 0,74-0,93 | NS |
| S VLDL C | 0,69 |  |  |  |  |  |  |  |  |  | 0,83 | 1,20 | 0,74-0,93 | NS |
| Ala |  |  |  |  |  | 0,62 |  |  |  |  | 0,82 | 1,22 | 0,72-0,93 | NS |
| XS VLDL P | 0,81 |  |  |  |  |  |  |  |  |  | 0,82 | 1,22 | 0,73-0,92 | NS |
| S LDL CE % | 0,90 |  |  |  |  |  |  |  |  |  | 0,81 | 1,23 | 0,73-0,80 | 0.009 |
| TotCho | 0,63 |  |  |  |  |  |  |  |  |  | 0,81 | 1,23 | 0,71-0,91 | NS |
| S LDL P | 0,94 |  |  |  |  |  |  |  |  |  | 0,8 | 1,25 | 0,71-0,89 | 0.007 |
| M LDL P | 0,97 |  |  |  |  |  |  |  |  |  | 0,79 | 1,27 | 0,71-0,89 | 0.005 |
| S LDL C % | 0,84 |  |  |  |  |  |  |  |  |  | 0,79 | 1,27 | 0,72-0,86 | 7.0x10^-5^ |
| S LDL L | 0,95 |  |  |  |  |  |  |  |  |  | 0,79 | 1,27 | 0,70-0,88 | 0.002 |
| XS VLDL L | 0,84 |  |  |  |  |  |  |  |  |  | 0,79 | 1,27 | 0,70-0,89 | 0.015 |
| LA FA |  |  |  | 0,67 |  |  |  |  |  |  | 0,78 | 1,28 | 0,70-0,87 | 5.0x10^-4^ |
| M LDL L | 0,97 |  |  |  |  |  |  |  |  |  | 0,78 | 1,28 | 0,70-0,87 | 0.002 |
| M LDL PL | 0,89 |  |  |  |  |  |  |  |  |  | 0,78 | 1,28 | 0,69-0,88 | 0.004 |
| S LDL CE | 0,99 |  |  |  |  |  |  |  |  |  | 0,78 | 1,28 | 0,71-0,87 | 1.3x10^-4^ |
| XS VLDL PL | 0,93 |  |  |  |  |  |  |  |  |  | 0,78 | 1,28 | 0,70-0,88 | NS |
| HDL3C | 0,73 |  |  |  |  |  |  |  |  |  | 0,77 | 1,30 | 0,67-0,88 | 0.012 |
| L LDL P | 0,97 |  |  |  |  |  |  |  |  |  | 0,77 | 1,30 | 0,69-0,87 | 0.001 |
| M LDL CE | 0,99 |  |  |  |  |  |  |  |  |  | 0,77 | 1,30 | 0,69-0,85 | 5.3x10^-5^ |
| M VLDL CE | 0,43 |  |  |  |  |  |  |  |  |  | 0,77 | 1,30 | 0,69-0,85 | 5.3x10^-5^ |
| RemtC | 0,79 |  |  |  |  |  |  |  |  |  | 0,77 | 1,30 | 0,68-0,86 | 0.001 |
| S LDL C | 0,98 |  |  |  |  |  |  |  |  |  | 0,77 | 1,30 | 0,69-0,86 | 1.3x10^-4^ |
| S LDL PL | 0,82 |  |  |  |  |  |  |  |  |  | 0,77 | 1,30 | 0,69-0,87 | 0.002 |
| S VLDL CE | 0,78 |  |  |  |  |  |  |  |  |  | 0,77 | 1,30 | 0,69-0,86 | 0.001 |
| XS VLDL FC | 0,89 |  |  |  |  |  |  |  |  |  | 0,77 | 1,30 | 0,69-0,87 | 0.001 |
| FreeC | 0,89 |  |  |  |  |  |  |  |  |  | 0,76 | 1,32 | 0,68-0,86 | 5.0x10^-4^ |
| L LDL L | 0,98 |  |  |  |  |  |  |  |  |  | 0,76 | 1,32 | 0,68-0,85 | 2.5x10^-4^ |
| LDL C | 0,99 |  |  |  |  |  |  |  |  |  | 0,76 | 1,32 | 0,68-0,84 | 3.3x10^-5^ |
| M LDL C | 0,99 |  |  |  |  |  |  |  |  |  | 0,76 | 1,32 | 0,68-0,84 | 5.4x10^-5^ |
| M LDL CE % | 0,90 |  |  |  |  |  |  |  |  |  | 0,76 | 1,32 | 0,68-0,85 | 2.5x10^-4^ |
| M VLDL CE % | 0,48 |  | 0,55 |  |  |  |  |  |  |  | 0,76 | 1,32 | 0,68-0,85 | 2.5x10^-4^ |
| S LDL FC | 0,90 |  |  |  |  |  |  |  |  |  | 0,76 | 1,32 | 0,67-0,85 | 2.5x10^-4^ |
| FaW6FA |  |  |  | 0,61 |  |  |  |  |  |  | 0,75 | 1,33 | 0,67-0,83 | 7.8x10^-6^ |
| IDL FC | 0,96 |  |  |  |  |  |  |  |  |  | 0,75 | 1,33 | 0,67-0,83 | 1.4x10^-5^ |
| IDL P | 0,96 |  |  |  |  |  |  |  |  |  | 0,75 | 1,33 | 0,67-0,84 | 1.2x10^-4^ |
| IDL PL | 0,98 |  |  |  |  |  |  |  |  |  | 0,75 | 1,33 | 0,67-0,84 | 6.5x10^-5^ |
| L LDL C | 0,98 |  |  |  |  |  |  |  |  |  | 0,75 | 1,33 | 0,67-0,83 | 1.2x10^-5^ |
| L LDL CE | 0,98 |  |  |  |  |  |  |  |  |  | 0,75 | 1,33 | 0,67-0,84 | 2.3x10^-5^ |
| L LDL FC | 0,97 |  |  |  |  |  |  |  |  |  | 0,75 | 1,33 | 0,68-0,83 | 7.3x10^-6^ |
| LA | 0,69 |  |  | 0,55 |  |  |  |  |  |  | 0,75 | 1,33 | 0,67-0,84 | 2.5x10^-4^ |
| M LDL FC | 0,95 |  |  |  |  |  |  |  |  |  | 0,75 | 1,33 | 0,67-0,84 | 1.3x10^-4^ |
| IDL L | 0,96 |  |  |  |  |  |  |  |  |  | 0,74 | 1,35 | 0,66-0,82 | 1.8x10^-5^ |
| L LDL PL | 0,97 |  |  |  |  |  |  |  |  |  | 0,74 | 1,35 | 0,66-0,83 | 4.6x10^-5^ |
| EstC | 0,91 |  |  |  |  |  |  |  |  |  | 0,73 | 1,37 | 0,65-0,82 | 2.8x10^-5^ |
| FAw6 | 0,74 |  |  | 0,46 |  |  |  |  |  |  | 0,73 | 1,37 | 0,64-0,82 | 5.0x10^-5^ |
| M LDL C % | 0,83 |  |  |  |  |  |  |  |  |  | 0,73 | 1,37 | 0,66-0,82 | 1.6x10^-6^ |
| M VLDL C% | 0,49 |  | 0,55 |  |  |  |  |  |  |  | 0,73 | 1,37 | 0,66-0,82 | 1.6x10^-6^ |
| SerumC | 0,91 |  |  |  |  |  |  |  |  |  | 0,73 | 1,37 | 0,65-0,83 | 5.1x10^-5^ |
| PUFAFA |  |  |  | 0,58 |  |  |  |  |  |  | 0,73 | 1,37 | 0,65-0,81 | 6.4x10^-7^ |
| L LDL CE % | 0,87 |  |  |  |  |  |  |  |  |  | 0,72 | 1,39 | 0,65-0,80 | 2.8x10^-8^ |
| PUFA | 0,74 |  |  | 0,42 |  |  |  |  |  |  | 0,72 | 1,39 | 0,64-0,82 | 3.9x10^-5^ |
| S VLDL C % | 0,51 |  |  |  |  |  |  |  |  |  | 0,72 | 1,39 | 0,64-0,81 | 3.0x10^-6^ |
| S VLDL CE % | 0,50 |  |  |  |  |  |  |  |  |  | 0,72 | 1,39 | 0,64-0,80 | 9.9x10^-7^ |
| XS VLDL C% |  | -0,62 |  |  |  |  |  |  |  |  | 0,72 | 1,39 | 0,64-0,80 | 2.4x10^-7^ |
| L LDL C % | 0,79 | -0,55 |  |  |  |  |  |  |  |  | 0,71 | 1,41 | 0,62-0,81 | 2.8x10^-5^ |
| XS VLDL CE% |  | -0,61 |  |  |  |  |  |  |  |  | 0,71 | 1,41 | 0,63-0,78 | 1.1x10^-8^ |
| IDL C | 0,96 |  |  |  |  |  |  |  |  |  | 0,7 | 1,43 | 0,63-0,79 | 1.2x10^-7^ |
| IDL CE | 0,94 |  |  |  |  |  |  |  |  |  | 0,69 | 1,45 | 0,62-0,78 | 6.4x10^-8^ |
| XS VLDL C | 0,89 |  |  |  |  |  |  |  |  |  | 0,68 | 1,47 | 0,61-0,77 | 3.4x10^-8^ |
| XS VLDL CE | 0,85 |  |  |  |  |  |  |  |  |  | 0,65 | 1,54 | 0,58-0,74 | 1.6x10^-10^ |
| Glutamine |  |  |  |  |  |  | 0,77 |  |  |  | 0,65 | 1,54 | 0,58-0,74 | 3.1x10^-10^ |
| IDL CE% |  | -0,81 |  |  |  |  |  |  |  |  | 0,62 | 1,61 | 0,56-0,70 | 1.2x10^-13^ |
| TotPG | 0,55 |  |  |  |  |  |  |  |  |  | 0,62 | 1,61 | 0,53-0,72 | 1.2x10^-7^ |
| IDL C % | 0,45 | -0,83 |  |  |  |  |  |  |  |  | 0,61 | 1,64 | 0,54-0,70 | 2.6x10^-12^ |

**Supplementary Figure 1** Fatty Acid Chain Length and OA progression


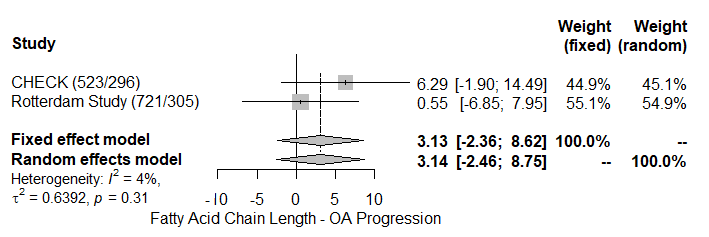

Supplement: Supplementary file 1 — Supplementary Information. [file 41598_2020_71811_MOESM1_ESM.docx]
